# Cytokines and related signaling pathways in traumatic brain injury

**DOI:** 10.3389/fimmu.2026.1738589

**Published:** 2026-02-11

**Authors:** Lihong Zhu, Siyun Huang, Weiqiang Chen, Kangsheng Li, Jiangtao Sheng

**Affiliations:** 1 Department of Microbiology and Immunology, Shantou University Medical College, Shantou, Guangdong, China; 2 The Department of International Medical Services, the Affiliated Cancer Hospital of Shantou University Medical College, Shantou, Guangdong, China; 3 Department of Neurosurgery, First Affiliated Hospital of Shantou University Medical College, Shantou, Guangdong, China

**Keywords:** cytokines, neuroinflammation, secondary injury after traumatic brain injury, signaling pathway, traumatic brain injury

## Abstract

Traumatic brain injury (TBI) represents a critical public health challenge with profound consequences for patients’ neurological function and quality of life. The delayed secondary injuries following TBI can lead to devastating long-term sequelae encompassing cognitive deficits, emotional disturbances, post-traumatic epilepsy, and neurodegeneration manifested as Alzheimer’s disease or Chronic Traumatic Encephalopathy (CTE). Emerging evidence highlights neuroinflammation as a pivotal mechanism driving secondary injury progression, establishing it as a prime therapeutic target in TBI management. Central to this process is the dysregulated cytokine release and associated signaling cascades that orchestrate neuroinflammatory responses. The pathological persistence of neuroinflammation arises from chronic glial activation and sustained immune cell infiltration following TBI. This review systematically examines recent advances in understanding cytokine dynamics and their regulatory pathways across different temporal phases of TBI-induced neuroinflammation. Notably, cytokines exhibit temporal functional pleiotropy - the same inflammatory mediators may exert diametrically opposed effects during acute (<24h), subacute (1-7d), and chronic (>7d) post-injury phases. This temporal dichotomy underscores the critical importance of precision timing when implementing cytokine-targeted therapies. Our comprehensive analysis integrates current clinical, preclinical and basic research evidence to illuminate potential mechanisms underlying TBI-associated neuropathology. We propose that multi-modal therapeutic strategies should combine spatiotemporal regulation of cytokine activity with pathway-specific interventions. This approach could potentially disrupt the self-perpetuating cycle of neuroinflammation while preserving beneficial reparative functions. The synthesis presented herein provides a framework for developing chronotherapeutic interventions against TBI-related neural dysfunction.

## 1 Introduction

Traumatic brain injury (TBI) refers to neurological dysfunction caused by external mechanical forces acting on the brain, typically resulting from head impacts due to falls, traffic accidents, sports injuries, physical abuse, or explosive blasts. These external forces induce primary mechanical damage that evolves into complex secondary biochemical responses (1, 2). With over 50 million annual cases worldwide, TBI presents substantial global health challenges - in China alone, the incidence rate reaches approximately 0.013% (3, 4).

As a dynamically evolving pathology, TBI progression involves two key phases: 1) The primary injury phase characterized by immediate mechanical tissue disruption 2) The secondary injury phase developing through biochemical cascades activated by the initial trauma. Acute presentations of TBI include intracranial hemorrhage, cerebral edema, and elevated intracranial pressure, which may progress to chronic neurological sequelae such as epilepsy, movement disorders, and dementia (5–7).

.These pathological changes significantly impair patients’ cognitive function, emotional regulation, and quality of life (8–10).

**Figure 1:**
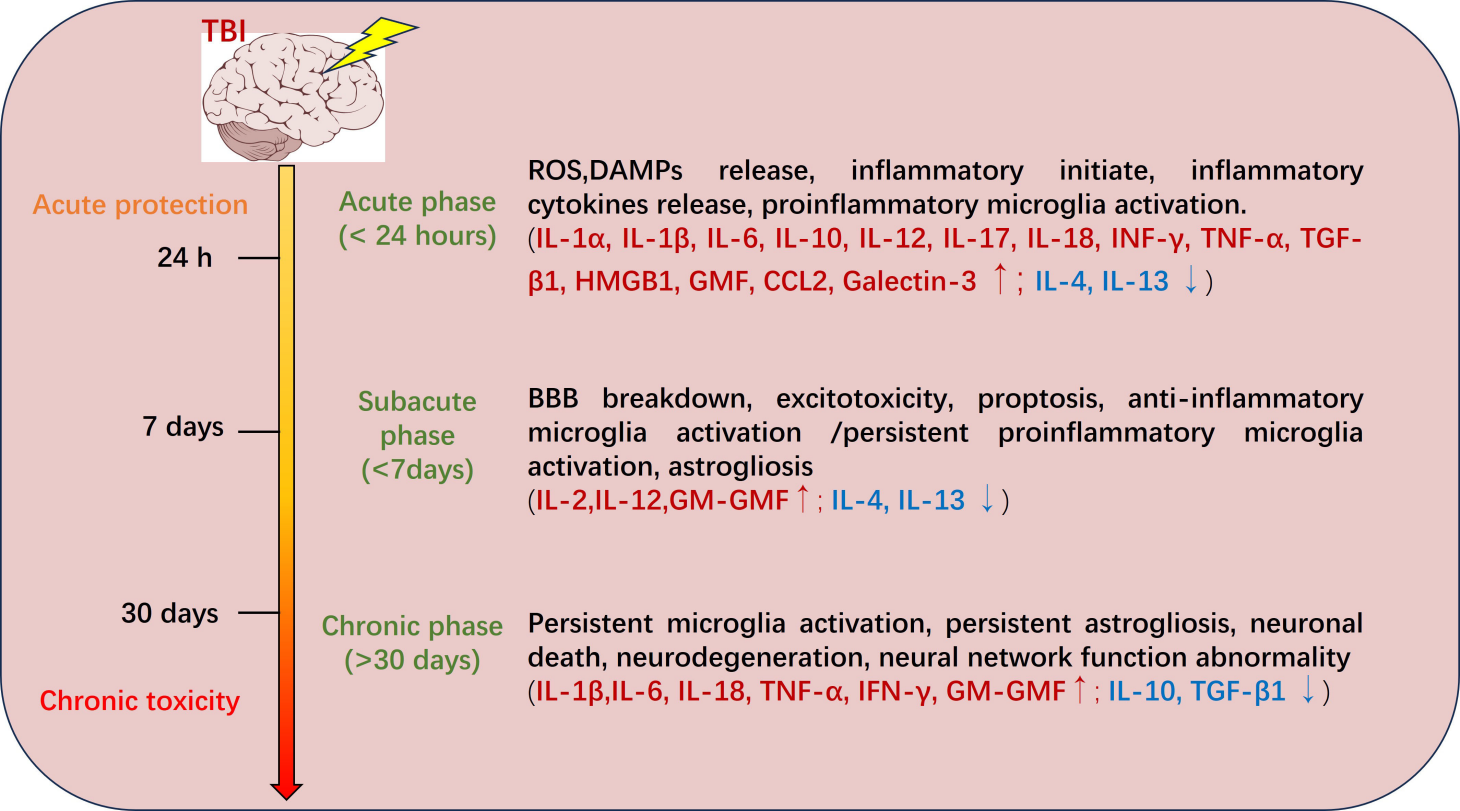
The development of traumatic brain injury as time progresses.

The molecular mechanisms underlying secondary injury in traumatic brain injury (TBI) involve coordinated pathological processes, which collectively drive progressive neurological deterioration across distinct temporal phases (**Figure 1**) (11). *The acute phase (<24 hours post-injury)* is characterized by rapid onset of inflammation triggered by reactive oxygen species (ROS) and damage-associated molecular patterns (DAMPs) release from injured cells, leading to proinflammatory microglia activation and cytokine storm. Key upregulated cytokines (e.g., IL-1α, IL-1β, IL-6, IL-12, IL-17, IL-18, IFN-γ, TNF-α, HMGB1, GM-CSF, CCL2, Galectin-3) orchestrate neuroprotective immune surveillance and debris clearance while risking excitotoxicity (12, 13), but this is compounded by early downregulation of anti-inflammatory/type 2 cytokines such as IL-4 and IL-13—often undetectable or markedly reduced in CSF, serum, and brain tissue—which limits counter-regulatory signaling and allows unchecked pro-inflammatory dominance, exacerbating excitotoxicity, BBB disruption, and acute neuronal loss (14–16). *In the subacute phase (1–7 days)*, persistent inflammation exacerbates secondary insults such as BBB breakdown, excitotoxicity, and pyroptosis, accompanied by mixed microglial activation and astrogliosis. Dominant cytokines (e.g., IL-2, IL-12, GM-CSF) modulate immune recruitment and partial resolution but potentially amplify damage through pathways like NF-κB/MAPK (17, 18), while sustained downregulation of IL-4 and IL-13, emerging IL-10 insufficiency, and relative TGF-β1 reduction delay inflammation resolution and aggravate edema and glial scarring (14, 15, 19). *The chronic phase (>30 days)* features sustained microglia and astrogliosis, leading to neuronal death, neurodegeneration, and neural network abnormalities, perpetuated by upregulated cytokines (e.g., IL-1β, IL-6, IL-18, TNF-α, IFN-γ, GM-CSF) that shift from reparative to maladaptive roles (20, 21) and by downregulation/exhaustion of resolving cytokines such as IL-10 and TGF-β1—evident in reduced serum IL-10 in moderate-to-severe TBI patients (~35 days post-injury), with even lower levels in those with post-traumatic confusional state—which fails to suppress persistent microglial activation, perpetuating chronic neuroinflammation, impaired sleep efficiency, and long-term sequelae like cognitive deficits and tauopathy (15, 19, 22–24). Chronic neuroinflammation emerges as a pivotal self-perpetuating process, driven by sustained inflammatory mediator release, pathological microglial activation, and cytokine imbalance that creates a pro-degenerative microenvironment.

## 2 Pathological diversity of TBI and the corresponding preclinical models

**Figure 2:**
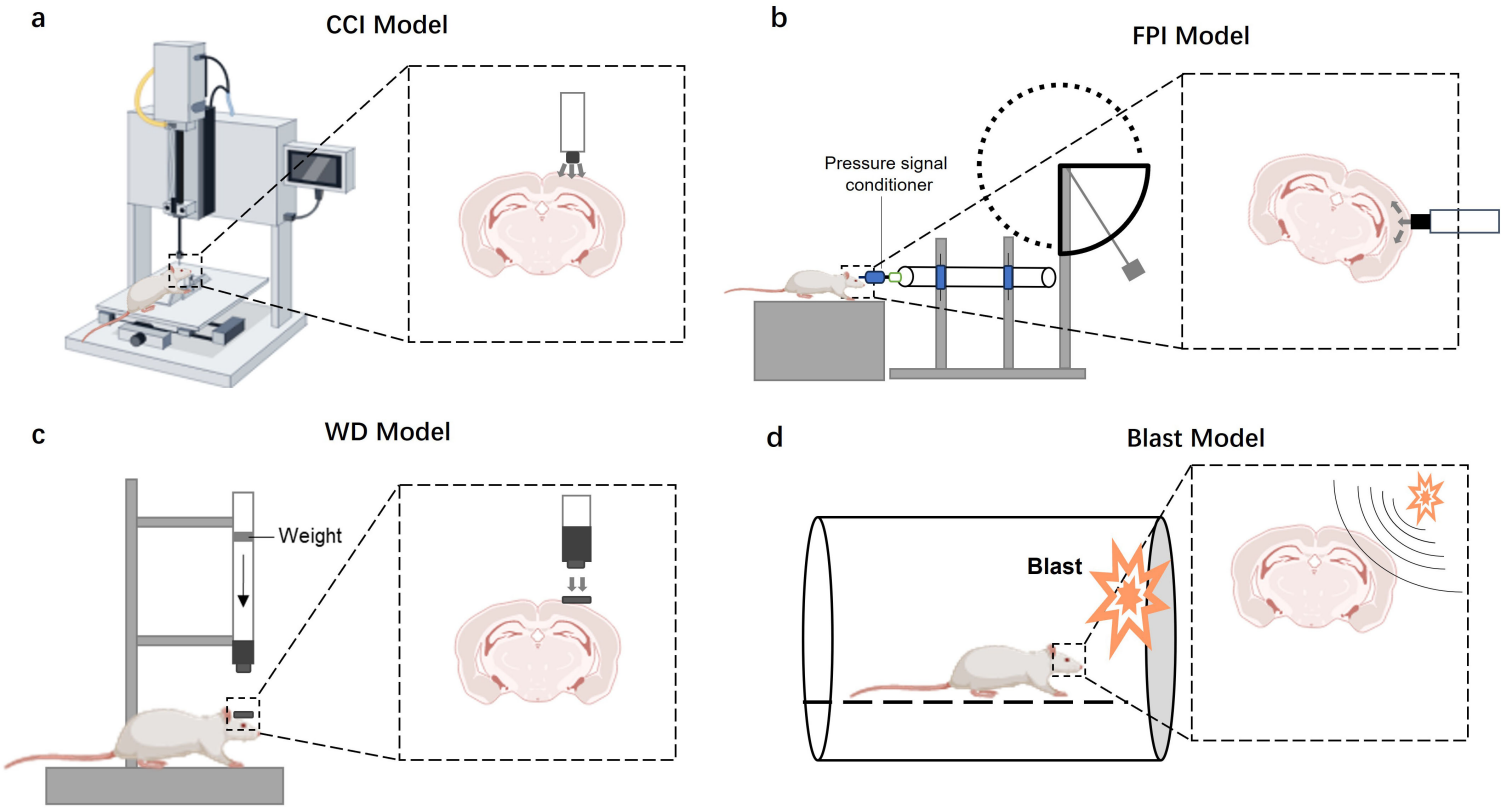
TBI models in mice. **(a)** CCI model: A pressure or electromagnetic controller is used to drive the striker to hit the brain. The severity of the injury is adjusted by controlling the rate, depth and dwell time of the percussion. **(b)** FPI model: Fluid pressure is delivered to the brain tissue by striking a circular column of fluid with a pendulum. The severity of the injury is regulated by controlling the pulse pressure by adjusting the height of the pendulum drop. **(c)** WD model: The pressure generated by the free fall of a heavy object passes through the cylindrical shape of the catheter and strikes the dura mater of the mouse. The severity of the injury is adjusted by varying the amount of pressure by adjusting the mass of the weight and the height of the fall. **(d)** Blast model: Shock waves caused by explosions result in head trauma. The severity of the injury is controlled by varying the size of the shock wave by adjusting the distance and intensity of the blast source.

Traumatic brain injury (TBI) is a clinically heterogeneous condition, typically stratified into mild, moderate, or severe categories based on metrics such as the Glasgow Coma Scale (GCS), duration of post-traumatic amnesia, and neuroimaging findings (25, 26). Anatomically, TBI manifests in diverse forms, including focal injuries like cerebral contusions and penetrating wounds, as well as diffuse injuries such as diffuse axonal injury (DAI) and concussion. To dissect the complex mechanisms of TBI and develop effective therapeutics, a variety of preclinical animal models have been established, each designed to recapitulate specific aspects of human pathology (27). A critical understanding of these models, including their strengths and limitations, is indispensable for translational research. Here, we discuss the most widely used TBI models, comparing their methodologies and clinical relevance (**Figure 2**; **Table 1**).

**Table 1:** Animal Models of TBI.

Model	Trigger	Species	Degree of injury	Type of injury	Strengths	Weaknesses
CCI model	Direct impact on exposed cortex with a pneumatic/electromagnetic piston.	Mice, rat	Controlled by impactor depth, velocity, and dwell time.	Focal cortical contusion with localized axonal injury and neuroinflammation.	High reproducibility and precise control of injury location and severity.	Craniotomy is required.
FPI model	Fluid pressure pulse delivered to the dura mater.	Mice, rat	Controlled by the magnitude of the pressure pulse.	Mixed focal and diffuse injury, including contusion, hemorrhage, and widespread axonal damage.	Models a complex injury pattern; highly reproducible.	Craniotomy is required.
WD model	A weight is dropped onto the intact skull.	Rat, Mice	Controlled by weight and drop height.	Closed-head contusion with potential for diffuse injury from inertial forces.	Clinically relevant closed-head model; no craniotomy.	Low reproducibility; risk of skull fracture.
Penetrating and ballistic brain injury model	A projectile breaches the skull and penetrates brain tissue.	Rat, Pig	Controlled by projectile size and velocity.	Penetrating wound with direct tissue laceration and cavitation.	Models specific human trauma (e.g., gunshot wounds).	Lack of standardized protocols.
Diffuse injury model	Sudden, rapid head rotation without direct impact.	Rat, Mice	Controlled by the magnitude of acceleration/rotation.	Widespread diffuse axonal injury (DAI) with minimal focal pathology.	Best model for concussion and DAI; mimics inertial forces.	Pathology can be subtle; lacks standardization.

CCI model, Controlled Cortical Impact model; FPI model, Fluid Percussion Injury model; WD model: Weight Drop model.

### 2.1 Models of focal brain injury

Focal injuries are characterized by localized damage, often resulting from direct impact. The Controlled Cortical Impact (CCI) model is a gold standard for inducing a reproducible focal contusion. In this model, a craniotomy is performed on an anesthetized animal, and a pneumatic or electromagnetic impactor piston is propelled onto the exposed dura mater (**Figure 1a**). The key advantage of the CCI model is its high degree of controllability; injury severity can be precisely titrated by adjusting parameters like impactor tip size, velocity, depth, and dwell time. This results in a highly reproducible cortical lesion with features resembling human contusions, including tissue loss, subdural hematomas, and axonal injury, making it ideal for mechanistic studies and therapeutic screening (28). However, its primary limitation is the requirement for a craniotomy, an invasive procedure that introduces its own inflammatory artifacts and does not mimic closed-head injuries.

The Fluid Percussion Injury (FPI) model is another widely used method that can produce both focal and diffuse injury components. It involves delivering a fluid pressure pulse onto the dura mater, either centrally (midline FPI) or laterally (lateral FPI), via a craniotomy (**Figure 1b**). The pressure pulse, generated by a pendulum striking a fluid-filled reservoir, causes rapid brain deformation. The severity is controlled by adjusting the height of the pendulum drop, which determines the magnitude of the fluid pulse. FPI effectively models a combination of contusion, subdural hematoma, and subarachnoid hemorrhage, and is extensively used in studies of post-traumatic epilepsy and neuroinflammation (29). Similar to CCI, its main weakness is the necessity of a craniotomy.

### 2.2 Models of closed-head and diffuse injury

To better simulate the biomechanics of falls or traffic accidents, closed-head injury models are employed. The Weight Drop (WD) model, also known as the Marmarou model, is a classic example. This model involves a free-falling weight striking the skull of an animal, which can be either fixed or allowed to move freely to generate rotational forces (**Figure 1c**). Unlike CCI or FPI, the WD model typically does not require a craniotomy, making it a more clinically relevant model for closed-head contusions. Injury severity is modulated by the weight and drop height. While this model more closely mimics the biomechanics of human TBI, its major drawback is lower reproducibility and a higher risk of skull fractures and uncontrolled secondary impacts, which can increase experimental variability (30).

Diffuse brain injuries, often resulting from rapid acceleration-deceleration forces seen in car accidents or sports, are primarily modeled using rotational injury paradigms. The Diffuse Injury model (e.g., impact acceleration model) induces widespread axonal injury by subjecting the animal’s head to sudden, rapid rotation without direct impact (**Table 1**). This model is crucial for studying the pathophysiology of DAI and the long-term consequences of concussion, such as Chronic Traumatic Encephalopathy (CTE), as it effectively simulates the shearing forces that damage axons throughout the white matter (27, 29).

### 2.3 Models simulating specific TBI scenarios

Certain models are tailored to specific real-world TBI scenarios. Penetrating and Ballistic Injury Models are designed to replicate injuries from projectiles like bullets or shrapnel, which are particularly relevant in military and forensic contexts. These models involve a foreign object breaching the skull and directly damaging brain tissue, with injury severity depending on the projectile’s size and velocity (31). Blast Injury Models use shock waves generated by explosions to simulate military-related injuries (**Figure 1d**). These models are unique in that they produce a complex injury pattern involving not only the primary blast wave but also secondary and tertiary effects from flying debris and body displacement. The severity is controlled by the intensity of the blast and the distance from the source. Both ballistic and blast models are critical for understanding the specific neuropathology seen in military personnel, but they often lack standardized protocols, which can complicate inter-study comparisons (32).

In summary, the selection of an appropriate TBI model is a critical decision that depends on the specific research question. While focal models like CCI and FPI offer high reproducibility for studying localized contusions, closed-head and diffuse injury models provide greater clinical relevance for common TBI scenarios. A thorough understanding of each model’s strengths and weaknesses, as outlined in **Table 1**, is essential for advancing our understanding of TBI pathophysiology and developing effective therapies.

## 3 Main cytokines in TBI

Cytokines play a central role in orchestrating the neuroinflammatory response that drives secondary injury in traumatic brain injury (TBI). After the initial mechanical trauma, damage-associated molecular patterns (DAMPs) from necrotic cells activate resident microglia, astrocytes, and infiltrating peripheral immune cells, leading to rapid cytokine production and release into the brain parenchyma, cerebrospinal fluid (CSF), and serum. This cytokine cascade fuels neuroinflammation, which has dualistic effects: acutely, pro-inflammatory cytokines (e.g., IL-1α/β, IL-6, IL-12, IL-17, IL-18, TNF-α, HMGB1) promote essential debris clearance, neutrophil recruitment, and immune surveillance but risk exacerbating edema, blood-brain barrier (BBB) disruption, excitotoxicity, and neuronal apoptosis; conversely, anti-inflammatory or resolving cytokines (e.g., IL-2, IL-4, IL-10, TGF-β1, GM-CSF) counteract excessive inflammation, support Treg expansion, glial reprogramming, and tissue repair (Simon et al., (22); Alam et al., (204); Piancone et al., (19)). Dysregulated balance—typically prolonged pro-inflammatory dominance with insufficient resolving signals—sustains chronic microglial/astrocyte activation, oxidative stress, and progressive neurodegeneration, contributing to long-term complications such as post-traumatic confusional state, cognitive impairment, and epilepsy. An overview of the primary cytokines involved, including their sources, temporal roles, and functional classification, is provided in **Table 2**.

**Table 2:** An overview of the main cytokines involved in traumatic brain injury.

Cytokines	Classification	Mechanism		Acute Phase Role	Chronic Phase Role	References
		Funding	Clarification			
IL-1α	Biomarker	15 patients: Higher IL-1α levels in 24h.	Clinical, age > 16 years	Pro-inflammatory (neurotoxic): Promotes neutrophil recruitment, BBB disruption, and early inflammation; exacerbates injury.	Limited data	Perez-Polo et al., 2016
	Treatment	Blocking IL-1α binding to IL-1R improves the extent of neuropathologic injury after TBI.	FPI model in rats, sTBI			
IL-1β	Biomarker	53 patients with sTBI: Higher IL-1β levels in 24h.	Clinical, age 18-50 years, mTBI, male (62.3%), motor vehicle accidents (37.7%)	Pro-inflammatory (neurotoxic): Drives acute neutrophil infiltration, BBB permeability, and cytokine storm; worsens edema and apoptosis.	Sustained elevation linked to chronic neuroinflammation	Vedantam et al, 2021; Ozen et al, 2020
	Treatment	Neutralizing IL-1β attenuates microglia activation and prevents the loss of neuron.	cFPI model in mice			
IL-2	Treatment	Injection of IL-2/IL-2C increases the number of Tregs, diminishs microgial cell activity and reduces the levels of pro-inflammations.	CCI model in mice, moderate TBI (1.5-mm-deep)	Limited; data	Neuroprotective: Promotes Treg expansion, reduces chronic inflammation; deficiency exacerbates neurotoxic astrocyte activation.	Gao et al, 2017
		Injection of IL-2/IL-2Ab increases the number of Tregs and enhances immunomodulatory function.	Ischemic model in mice			Zhang et al, 2018
	Pathology	Neutralisation of IL-2 leads to Treg cell deficiency to inhibit neurotoxic astrocyte activation.	Ischemic model in mice			Ito et al, 2019
IL-4	Biomarker	93 patients with TBI: Higher IL-4 in serum in 24h.	Clinical, age 15-83 years, male (80%), fall (56%) and motor vehicle accident (39%)	Neuroprotective: Attenuates early inflammatory response, inhibits microglia/astrocyte overactivation.	Sustained role in resolving chronic inflammation; promotes tissue repair and functional recovery.	Johnson et al., 2022
		IL-4 is secreted and produced by neuronsin 24h.	Middle cerebral artery (MCA) occlusion model			Zhao et al., 2015
	Pathology	IL-4 attenuates the inflammatory response and inhibits the abnormal activation of microglia and astrocytes.	CCI model in rat			Radpour et al., 2023
	Treatment	Exogenous IL-4 accelerates inflammatory regression and ameliorates post-TBI dysfunction and tissue damage.	SCI model in mice			(Francos-Quijorna et al., 2016
IL-6	Biomarker	53 patients with sTBI: Higher IL-6 levels in 24h.	Clinical, age 18-50 years, mTBI, male (62.3%), motor vehicle accidents (37.7%)	Pro-inflammatory (neurotoxic): Contributes to acute edema, BBB breakdown, and cytokine amplification; associated with worse early outcomes.	Dual/chronic pro-inflammatory: Linked to persistent gliosis and neurodegeneration; elevated in chronic TBI with poor prognosis.	Vedantam et al, 2021
		110 children with TBI: Higher IL-6 levels in 4h.	Clinical, age 5-12 years, 104 with mTBI and 6 with sTBI, sports(51%) and accidents including falls(42.3%)			Ryan et al, 2022
	Pathology	Pioglitazone attenuates neuroinflammation by inhibiting the PPARγ/NF-κB/IL-6 signalling pathway.	CCI model in rat, sTBI(5-mm-deep)			Deng et al, 2020
		Microglial IL-6 deficiency altered microglia and astrocyte activation markers and PPARγ kinetics to accelerate injury repair.	Cryolesion of the somatosensorial cortex model in mice			Sanchis et al, 2020
	Treatment	IL-6 stimulates the regeneration of microglia.	CCI model in mice, mTBI(1-mm-deep)			Willis et al, 2020
IL-10	Biomarker	166 patients with TBI: Higher IL-10 levels in 24h.	Clinical, age <16 years, 33 with moderate TBI and 133 with sTBI, male(74.7%)	Neuroprotective (acute): Suppresses early pro-inflammatory cytokines, reduces oxidative stress.	Neuroprotective (chronic): Promotes resolution of inflammation, supports long-term recovery; elevated in favorable outcomes.	Di Battista et al, 2016
	Pathology	IL-10/STAT3 signaling pathway activate to inhibite the production of ROS as anti-inflammatory response.	CCI model in mice, moderate TBI(2-mm-deep)			Barrett et al, 2017
	Treatment	Electroacupuncture treatment leads to increase IL-10 levels and inhibits macrophage autophagy through the blockade of the AMPK / mTOR signaling pathway.	Neuropathic pain model in rats			Wu et al, 2018
IL-12	Biomarker	32 patients with sTBI: Higher IL-12 levels in acute stage.	Clinical, age >18 years, male(75%)	Pro-inflammatory (neurotoxic): Enhances early inflammation, NK cell suppression leading to secondary insults.	Sustained neurotoxic: Contributes to chronic immune dysregulation; limited long-term data.	Roquilly et al, 2017
		Higher IL-12 levels at 3 days after stroke.	Ischemic model in mice			Lee et al, 2016
	Pathology	IL-12 attenuates IFN-γ production and cytotoxicity capacity of NK cells, leading to further inflammation.	Ischemic model in mice			Lee et al, 2016
IL-17	Biomarker	Higher IL-17 levels in acute phase.	Diffuse TBI model in rats	Pro-inflammatory (neurotoxic): Promotes acute neuronal apoptosis.	limited data.	Amiresmaili et al, 2020
	Pathology	IL-17 favour inflammatory microglia and exacerbate the inflammatory response.	CCI model in mice, mTBI(1-mm-deep)			Abou-El-Hassan et al, 2023
	Treatment	Propofol inhibits IL-17 production by miR-145-3p / NFATc2 / NF-κB signalling pathway.	CCI model in rats, moderate TBI			Cui et al, 2021
IL-18	Biomarker	93 patients with TBI: Higher IL-18 levels in 12h.	Clinical, age 15-83 years, male (80%), fall (56%) and motor vehicle accident (39%)	Pro-inflammatory (neurotoxic): Drives acute inflammasome activation, worse 6-month outcomes; associated with poor prognosis.	Neurotoxic (chronic): Persistent elevation linked to ongoing inflammation and neurodegeneration	Vedantam et al, 2021
		16 patients with TBI: Higher IL-18 levels in chronic phase.	Clinical, 16 with sTBI			Ciaramella et al, 2014
	Treatment	Knockdown of IL-18 resulted in attenuated activation of astrocytes and microglia.	CCI model in mice, mTBI (1-mm-deep)			Dong et al, 2020
IFN-γ	Biomarker	Post-mortem human brain: Higher IFN-γ levels.	Clinical	Pro-inflammatory (neurotoxic): Promotes acute microglial activation and cytokine storm.	Sustained neurotoxic: Contributes to chronic neurodegeneration and poor outcomes.	Roselli et al, 2018
	Pathology	IFN-γ enhances microglia initiation and response.	CCI model in mice			Wangler & Godbout, 2023
TNF-α	Biomarker	Higher TNF-α levels in acute stage.	CCI model in rats, repetitive mTBI	Dual (neuroprotective/neurotoxic): Acute exacerbation of BBB disruption and apoptosis; some protective effects.	Neurotoxic (chronic): Sustained inflammation leading to neurodegeneration.	Qin et al, 2017
	Pathology	TNF-α induces the synthesis of metalloproteinase via Ca2+ / CAMK II / ERK / NF-κB signaling.	In vitro BBB model			Ding et al, 2019
	Treatment	TNF-α inhibits abnormal microglia activation and suppresses inflammation.	In vitro, hNPCs			Kim et al., 2020
TGF-β1	Biomarker	35 patients with TBI: Higher TGF-β1 levels in 24h.	Clinical, male (74.3%)	Dual (neuroprotective/neurotoxic): Acute promotion of inflammation/apoptosis; some glial modulation.	Dual: Chronic fibrosis/gliosis; promotes repair via ERK/NF-κB inhibition.	Li et al, 2021
	Pathology	TGF-β1 mediates the microglia transition from neurotoxicity to neuroprotection.	CCI model in rats, sTBI (3-mm-deep)			Zhao et al, 2020a
		TGF-β1 promotes IL-1 and TNF-a production, exacerbating apoptosis and oxidative stress.	FPI model in rats, sTBI			Patel et al., 2017
	Treatment	Exogenous TGF-β1 modulates the ERK1 / 2 and NF-κB signaling pathways to attenuate microglia-mediated inflammation.	LPC-induced demyelinating model in mice			Xie et al, 2022b
HMGB1	Pathology	HMGB1 induces the activation of pro-inflammatory microglia and pro-inflammatory cytokines through the RAGE-nuclear factor-κB pathway.	Spinal cord injury model in rats	Neurotoxic: Drives acute inflammation and cytokine release.	Sustained neurotoxic: Contributes to chronic neurodegeneration.	Fan et al, 2020
GM-CSF	Biomarker	Higher GM-CSF levels from the third to the sixtieth day.	CCI model in rats, mTBI (2-mm-deep)	Neuroprotective: Acute suppression of inflammation.	Neuroprotective (chronic): Sustained Treg regulation and recovery.	Vinh To et al, 2022
	Treatment	GM-CSF suppresses pro-inflammatory factor expression.	CCI model in Yorkshire swines, sTBI (8-mm-deep)			Williams et al, 2020
		Exogenous GM-CSF regulates the number of Tregs and inhibits the proliferation of reactive microglia.	MPTP-intoxicated model in mice			Olson et al, 2020
CCL2	Biomarker	92 patients with TBI: Higher CCL2 levels in 24h.	Clinical, 28 with moderate TBI and 64 with sTBI, male (70.7%)	Neurotoxic: Promotes acute glial reactivity and gene expression.	limited data.	Chen et al, 2023
	Treatment	Knockdown of CCL2 reduces inflammatory gene expression and reactive glial cells and reverses apoptosis.	CCI model in mice			Gyoneva & Ransohoff, 2015
GMF	Pathology	Increased levels of GMF activate astrocytes, exacerbating oxidative stress and neuronal death.	In vitro scratch model	Neurotoxic: Acute oxidative stress and neuronal death.	Neurotoxic (chronic): Sustained gliosis and cytokine release.	Ahmed et al., 2020b
	Treatment	Mutation or knockdown of GMF attenuated gliosis, and reduction of IL-1β, IL-6 and TNF-α contributed to motor function recovery.	CCI model in mice			Selvakumar et al., 2020 Ahmed et al., 2020a
Galectin-3	Biomarker	Galectin-3 is released from brain parenchyma to cerebrospinal fluid in the acute phase	CCI model in mice	Neurotoxic: Acute promotion of inflammation and neuronal damage.	limited data.	Venkatesan et al., 2010
	Pathology	Galectin-3 exacerbated IL-1β, IL-6, TNFα and NOS2 expression, promoting inflammation and damage to neurons.	CCI model in mice			Yip et al., 2017
	Treatment	Neutralization or knockdown of Galectin-3 significantly promotes neuroprotection in damaged cortical areas and hippocampal cell populations.	CCI model in mice			Yip et al., 2017

IL-1α: Interleukin-1α; IFN-γ: Interferon gamma ; TNF-ɑ: tumor necrosis factor α; TGF-β1:Transforming growth factor β 1; HMBG1: High mobility group protein 1; GM-CSF: granulocyte-macrophage colony-stimulating factor; CCL2: C C motif ligand 2; GMF: glia maturation factor.

### 3.1 The IL-1 family

The Interleukin-1 (IL-1) family, comprising IL-1α and IL-1β, represents a cornerstone of the innate immune response and is a pivotal driver of the initial neuroinflammatory cascade following TBI (33). While often grouped together, their distinct biological properties necessitate a separate yet integrated discussion.

IL-1α acts as a primary alarmin, uniquely positioned to signal immediate cellular stress. Unlike the strictly inducible IL-1β, IL-1α is constitutively expressed in an active precursor form within CNS-resident cells, particularly astrocytes, where it contributes to barrier homeostasis (8). Upon cell injury and necrotic release, this pre-formed IL-1α can directly engage the Type I IL-1 receptor (IL-1R1) to trigger a rapid inflammatory response without the need for inflammasome processing. This makes IL-1α a critical initiator of the earliest post-TBI inflammatory events.

In contrast, IL-1β is the archetypal inducible pro-inflammatory cytokine. Its production requires a two-step activation process: a priming signal upregulates pro-IL-1β transcription, followed by cleavage into its mature, secretable form by caspase-1 within the NLRP3 inflammasome (34). This tightly regulated process ensures that IL-1β is massively released only in response to significant danger signals. Clinically, elevated CSF levels of IL-1β correlate strongly with acute-phase inflammatory markers and injury severity (35). Mechanistically, IL-1β neutralization has been shown to mitigate microglial activation and protect vulnerable neuronal populations, such as parahippocampal interneurons (36, 37).

Despite their differences, both IL-1α and IL-1β converge on the IL-1R1 signaling axis, leading to synergistic pro-inflammatory effects. Studies using IL-1R1 knockout mice demonstrate superior neuroprotection compared to isoform-specific inhibition, highlighting their combined pathological impact (38). The activation of the IL-1/IL-1R1 axis orchestrates a multi-faceted assault on CNS integrity, including: 1) Inflammatory Amplification via NF-κB and MAPK pathways; 2) Barrier Disruption of the blood-brain, meningeal, and blood-retinal barriers (39); and 3) Leukocyte Trafficking into the CNS. This positions the IL-1R1 as a prime therapeutic target for mitigating acute secondary injury.

### 3.2 IL-2

While pro-inflammatory cytokines dominate the acute phase, endogenous counter-regulatory mechanisms are simultaneously activated (40, 41). Interleukin-2 (IL-2), traditionally known for its role in T cell proliferation, is emerging as a key player in promoting neuroinflammation resolution through its potent support of regulatory T cell (Treg) survival and function (42).

Preclinical studies have demonstrated that administration of IL-2/anti-IL-2 antibody complexes (IL-2C) significantly expands Treg populations in the injured brain. This expansion correlates with attenuated microglial activation, suppressed pro-inflammatory cytokine release (IL-1β, TNF-α), and enhanced neurovascular integrity (235). The therapeutic potential of this axis extends into the chronic phase; recent groundbreaking work shows that delayed, low-dose IL-2 (LD-IL-2) treatment can reverse chronic TBI sequelae, such as post-traumatic headaches and cognitive deficits, by replenishing meningeal Treg populations (43).

To overcome the challenges of systemic administration, innovative CNS-targeting strategies are being developed. An astrocyte-specific gene delivery system for local IL-2 overexpression has shown remarkable efficacy in suppressing reactive astrogliosis and improving functional outcomes across multiple CNS injury models (44). Collectively, these findings establish the IL-2/Treg axis as a promising therapeutic strategy for both acute attenuation and chronic resolution of neuroinflammation.

### 3.3 IL-4

Interleukin-4 (IL-4) is a pleiotropic cytokine that serves as a master regulator of the transition from pro-inflammatory responses to tissue repair. Elevated serum IL-4 levels in TBI patients suggest its active involvement in post-injury immune modulation (45). Its neuroprotective mechanisms are multifaceted, primarily centered on reprogramming the function of glial cells.

IL-4 is a potent driver of anti-inflammatory glial reprogramming. It directs microglia and macrophages away from a pro-inflammatory state (e.g., CD86+) towards a reparative phenotype (e.g., CD206+), which is crucial for clearing debris and promoting neural repair (15). It achieves this by upregulating anti-inflammatory mediators like IL-10 while suppressing pro-inflammatory cytokines like TNF-α (46). Furthermore, exogenous IL-4 attenuates reactive astrogliosis, a key component of the glial scar that impedes regeneration.

The therapeutic utility of IL-4 is conserved across various CNS injury models. In spinal cord injury, for instance, IL-4 treatment accelerates inflammation resolution and improves motor recovery (47). Excitingly, advanced delivery strategies are enhancing its therapeutic potential. Nanoparticle-encapsulated IL-4 has been shown to activate the PPARγ pathway specifically in oligodendrocyte precursor cells, promoting their differentiation and restoring white matter integrity post-TBI (48). These findings underscore IL-4’s role not just as an anti-inflammatory agent, but as a key mediator of CNS tissue repair, particularly in the context of white matter injury.

### 3.4 IL-6

Interleukin-6 (IL-6), a pleiotropic cytokine primarily secreted by macrophages, T lymphocytes, and stromal cells, orchestrates acute-phase protein synthesis. Under physiological conditions, IL-6 is virtually undetectable in the CNS. However, following traumatic brain injury (TBI), IL-6 is rapidly upregulated in microglia, astrocytes, and neurons, with elevated levels observed in cerebrospinal fluid and serum within hours post-injury (49). This cytokine promotes blood-brain barrier (BBB) dysfunction, exacerbates neurological deficits, and amplifies inflammatory cascades through STAT3 and MAPK pathways.

The PPARγ/NF-κB/IL-6 axis serves as a key regulatory mechanism for IL-6 production in TBI. Pharmacological activation of PPARγ with pioglitazone (10 mg/kg) suppresses NF-κB nuclear translocation, reduces IL-6 levels, and attenuates neuroinflammation and cerebral edema (50). Genetic evidence further supports this pathway: microglia-specific IL-6 deletion alters glial activation profiles and accelerates functional recovery via PPARγ-mediated repair mechanisms (51).

Notably, IL-6 exhibits context-dependent duality in TBI pathophysiology: In the acute phase, IL-6 exerts pro-inflammatory effects by driving neurotoxic inflammation through classical membrane-bound IL-6 receptor (IL-6R) signaling. Conversely, during the subacute phase, IL-6 displays neuroprotective effects by stimulating microglial reprogramming, enhancing neuronal survival, and improving cognitive outcomes through soluble IL-6R (sIL-6R) trans-signaling (52). This temporal dichotomy underscores the need to delineate stage-specific roles of IL-6 signaling—targeting its detrimental acute actions while preserving regenerative functions—to develop precision therapeutics for TBI.

### 3.5 IL-10

IL-10, an anti-inflammatory cytokine predominantly secreted by Th cells and macrophages, suppresses macrophage antigen-presenting capacity, downregulates pro-inflammatory cytokine production (e.g., TNF-α, IL-1β), and attenuates Th1-driven immune responses. Within the CNS, IL-10 exerts neuroprotection through microglial modulation: in animal models, IL-10 enhances β-endorphin expression in microglia, mediating antinociceptive effects and promoting neural repair (53).

In traumatic brain injury (TBI), IL-10 has neuroprotective effects through various mechanisms. Firstly, during the acute phase of TBI, IL-10 helps transition microglia to an anti-inflammatory M2 phenotype, reducing TNF-α and IL-1β secretion and promoting tissue preservation (54). Secondly, IL-10/STAT3 signaling inhibits NADPH oxidase (NOX)-dependent ROS generation, suppressing oxidative stress and neuroinflammation via NF-κB pathway inactivation (55). Lastly, IL-10 protects the blood-brain barrier (BBB) by preventing vascular endothelial cell apoptosis through STAT3-mediated Bcl-2 upregulation, maintaining BBB integrity in rodent TBI models (56).

In the clinical setting, IL-10 also has various potential applications. Serum IL-10 levels can be used as a prognostic biomarker for predicting TBI outcomes with high sensitivity (96%) and moderate specificity (50%) (57). Elevated IL-10, along with IL-6 and IL-8, can predict the development of acute respiratory distress syndrome in severe TBI patients (AUC = 0.84). IL-10 deficiency worsens post-TBI cognitive deficits and neuronal loss specifically in male mice, leading to increased hippocampal gliosis (58). These findings position IL-10 as both a therapeutic target (via STAT3 pathway modulation) and a clinically actionable biomarker for TBI management.

### 3.6 IL-12

Interleukin-12 (IL-12), a heterodimeric cytokine primarily secreted by activated antigen-presenting cells such as dendritic cells and macrophages, plays a pivotal role in driving Th1 immune responses and stimulating interferon-gamma (IFN-γ) production by T cells and natural killer (NK) cells. Elevated IL-12 levels are observed in both clinical traumatic brain injury (TBI) patients and experimental models during the early post-injury phase (59, 60). Therapeutic interventions such as hydrogen administration have been shown to mitigate neuroinflammation by restoring physiological IL-12 expression levels and suppressing microglial hyperactivation (61). Notably, IL-12 counteracts TBI-induced immunosuppression—a hallmark of severe TBI characterized by impaired NK cell cytotoxicity, reduced IFN-γ production, and T cell depletion—by enhancing NK cell function and indirectly revitalizing cellular immunity (60). However, the role of IL-12 in CNS injury exhibits context-dependent duality. While its immunostimulatory effects may confer neuroprotection, the IL-12p40 subunit, essential for IL-12 bioactivity, paradoxically exacerbates pathological damage in spinal cord injury models, as evidenced by improved functional recovery and reduced lesion volumes in IL-12p40-deficient mice (62). Whether this detrimental role extends to TBI remains unclear, underscoring the need to elucidate the mechanistic basis of IL-12’s dual effects in TBI pathogenesis and its potential as a therapeutic target.

### 3.7 IL-17

Interleukin-17 (IL-17), a pro-inflammatory cytokine predominantly secreted by T helper 17 (Th17) cells, exacerbates neuroinflammatory cascades by inducing granulocyte colony-stimulating factor (G-CSF) and chemokine production, thereby promoting inflammatory cell infiltration. This cytokine polarizes microglia toward a pro-inflammatory phenotype (CD68+/iNOS+), amplifies neuroimmune activation, and disrupts blood-brain barrier integrity through matrix metalloproteinase-9 (MMP-9) upregulation (63, 64). Temporospatial analyses reveal that IL-17 levels in both CNS tissues and systemic circulation escalate progressively from 6 hours to 7 days post-TBI, peaking at 72 hours (65)—a kinetic profile strongly correlating with secondary injury severity (r = 0.78, p < 0.01). Mechanistically, IL-17 drives neuroinflammation via NF-κB pathway activation, as evidenced by the therapeutic efficacy of IL-17 neutralization: Secukinumab (anti-IL-17A antibody) reduces phosphorylated p65 levels by 62% and attenuates neuronal apoptosis (66). Preclinical interventions further demonstrate that propofol-mediated IL-17 suppression restores Th17/Treg balance through the miR-145-3p/NFATc2/NF-κB axis, reducing cortical lesion volume by 38% and improving neurological scores in rodent TBI models (67). These findings collectively position IL-17 as a promising therapeutic target for early-phase TBI management through NF-κB pathway blockade.

### 3.8 IL-18

Interleukin-18 (IL-18), initially characterized for its role in driving interferon-γ (IFN-γ) production and orchestrating immune cell infiltration, serves as a pivotal mediator bridging innate and adaptive immunity by activating leukocytes/lymphocytes and amplifying inflammatory apoptosis (68). In traumatic brain injury (TBI), microglia-derived IL-18 undergoes caspase-1-dependent cleavage—a process triggering synaptic glutamate release and NLR-mediated neuroinflammation via pathogen/damage-associated molecular pattern (PAMP/DAMP) recognition through toll-like receptors (TLRs) (69, 70). Mechanistically, DAMP-TLR engagement post-TBI activates NLRP3, NLRP1, or AIM2 inflammasomes, facilitating IL-1β/IL-18 maturation while concurrently inducing pyroptosis—a lytic cell death marked by membrane pore formation and further cytokine spillage (71, 72). Temporally distinct from IL-1β’s acute proinflammatory dominance, IL-18 critically governs chronic neuropathology: elevated serum IL-18 levels correlate with poor long-term prognosis and drive persistent neuroinflammation linked to neuronal dysfunction and neurodegeneration (34, 73, 74). Therapeutic targeting of IL-18 with its endogenous antagonist IL-18 binding protein (IL-18BP) demonstrates delayed efficacy—neurological recovery emerges only after day 7 post-TBI, suggesting time-dependent modulation of IL-18 signaling cascades or compensatory pathway activation during acute phases.

**Figure 3:**
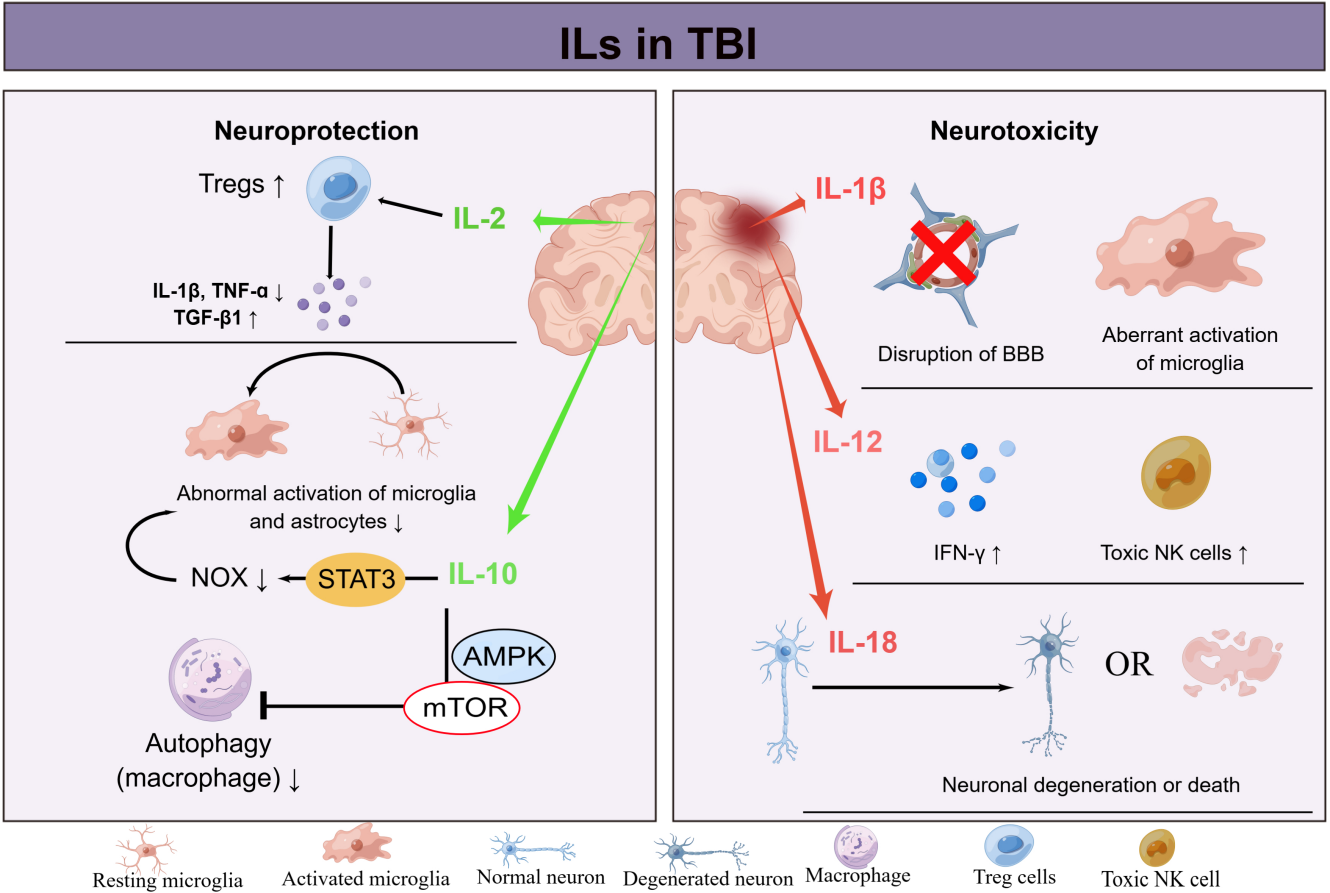
Interleukins play a role in the regulation of neuroinflammation following TBI. Exogenous IL-2 has been shown to enhance neuroprotection by increasing the population of regulatory T cells (Tregs) and suppressing microglial activity. Additionally, IL-2 reduces levels of pro-inflammatory cytokines IL-1β and TNF-α, while promoting the release of the anti-inflammatory factor TGF-β1. Elevated levels of IL-10 during the acute phase of TBI inhibit astrocyte activation through the suppression of NOX production via the activation of the STAT3 signaling pathway. Furthermore, IL-10 inhibits macrophage autophagy by blocking the AMPK/mTOR signaling pathway. Regarding neurotoxicity, increased levels of IL-1 have been shown to compromise the integrity of the blood-brain barrier, leading to the infiltration of peripheral immune cells and the release of inflammatory mediators that activate microglia. In contrast, IL-12 has been found to promote the differentiation of cytotoxic NK cells and the secretion of IFN-γ, thereby intensifying the inflammatory response. Additionally, elevated levels of IL-18 have been implicated in the perpetuation of chronic neuroinflammation, resulting in heightened neuronal apoptosis and dysfunction.

In summary, interleukins exhibit marked functional pleiotropy in TBI pathophysiology, with certain family members exerting neuroprotective effects while others drive neurotoxic inflammation (**Figure 3**). IL-2 promotes neuroprotection by expanding regulatory T cell (Treg) populations, suppressing microglial activation, reducing pro-inflammatory cytokines such as IL-1β and TNF-α, and enhancing anti-inflammatory TGF-β1 release. Similarly, IL-10 confers neuroprotection in the acute phase by inhibiting astrocyte activation via STAT3-mediated suppression of NOX production and by blocking macrophage autophagy through the AMPK/mTOR pathway. In contrast, IL-1 (encompassing IL-1α and IL-1β) exerts neurotoxicity by disrupting blood-brain barrier integrity, facilitating peripheral immune cell infiltration, and activating microglia to release inflammatory mediators. IL-12 intensifies inflammation by promoting cytotoxic NK cell differentiation and IFN-γ secretion, while IL-18 perpetuates chronic neuroinflammation, leading to sustained neuronal apoptosis and dysfunction. This temporal and contextual duality underscores the potential for targeted interleukin modulation to shift the balance toward resolution and repair in TBI.

### 3.9 Interferon-γ

Interferon-gamma (IFN-γ), a pleiotropic cytokine primarily secreted by T-lymphocytes and natural killer cells, is also endogenously produced by neurons and glial cells within the central nervous system (CNS), where it regulates glial proliferation, maturation, and synaptic network remodeling (75). Elevated IFN-γ levels are observed in both cerebrospinal fluid and plasma during acute traumatic brain injury (TBI), correlating with neuroimmune modulation and neuronal circuit dysregulation (76, 77). While IFN-γ drives neurotoxic microglial hyperactivation via the STING pathway—a mechanism linked to persistent cognitive deficits in murine TBI models (78)—it paradoxically exhibits neuroprotective properties in stab wound injury contexts, enhancing brain-derived neurotrophic factor (BDNF) secretion, reducing cortical neuron apoptosis, and promoting astroglial scar formation (79). This functional duality is further complicated by genetic studies showing contradictory outcomes, suggesting concentration-dependent and spatiotemporal regulation of IFN-γ signaling (77). Notably, mild TBI patients exhibit sustained IFN-γ elevation even 12 months post-injury, implicating chronic innate immune activation in long-term neurological sequelae (80). These conflicting observations underscore the need for rigorous investigation into IFN-γ’s temporal dynamics, dose-response relationships, and downstream effector mechanisms before considering its therapeutic targeting in TBI management.

### 3.10 Tumor necrosis factors-α

TNF-α is a pro-inflammatory cytokine secreted mainly by macrophages and monocytes, and plays an important role in neuroinflammation by activating several signaling pathways upon binding to its receptor. In a rat mild brain injury, TNF-α, were significantly increased in rat brain tissue, and neurodegeneration and induced astrocytosis in the hippocampus (81). Elevated levels of TNF-α, as well as other pro-inflammatory cytokines (IL-6, IL-10) and soluble intracellular adhesion molecule (sICAM-1), have been associated with poor prognosis following severe TBI in adult men patients (82). In the context of TBI, short-acting TNF-α activates the TAK1 phosphorylation-dependent JNK pathway to protect cells, while long-acting TNF-α induces ASK1 phosphorylation-dependent JNK pathway, ultimately leading to caspase-dependent apoptosis (49). In addition, TNF-α controls the synthesis of neurotrophin and stops calcium influx-induced neuronal death (83). TNF-α also induces the synthesis of metalloproteinase, a key constituent of the blood-brain barrier, via Ca2+/CAMK II/ERK/NF-κB signaling, thereby disrupting blood-brain barrier permeability and exacerbating the response (84). Zheng et al’s study revealed TNF-α derived from microglia activates downstream NF-κB/iNOS signaling, leading to neuroinflammation, oxidative stress, and further deterioration of TBI (85). Moreover, TNF-α has the potential to further aggravate nerve damage through by activating the p53-induced apoptosis signaling pathway (86). However, TNF-α may also exert protective effects on neuronal cell death. In a neonatal rat model of hypoxic-ischemic brain injury, TNF-α has been shown to have protective effects on neuronal cell death by changing the polarization of microglia and increasing the expression of neurotrophic factors. Studies using transplantation of TNF-α-pretreated human neural progenitor cells (hNPCs) have demonstrated regression of ischemic morphology and improvements in neurological function (87). Therefore, this neuronal stem cell therapy utilizing TNF-α may also have neuroprotective effects against nonischemic traumatic brain injury (TBI).

Tumor necrosis factor-alpha (TNF-α), a key pro-inflammatory cytokine predominantly secreted by macrophages and monocytes, exerts multifaceted roles in TBI pathophysiology through receptor-dependent activation of downstream signaling cascades. In rodent TBI models, elevated TNF-α levels correlate with hippocampal neurodegeneration and reactive astrogliosis (81). Clinically, sustained TNF-α elevation—alongside IL-6, IL-10, and sICAM-1—predicts poor outcomes in severe TBI patients (82).

Mechanistic studies reveal its dichotomous effects: Firstly, Neurotoxic pathways: Chronic TNF-α exposure activates ASK1/JNK/caspase-3 apoptosis axis, while acute signaling via TAK1/JNK confers transient cytoprotection (49). Microglial TNF-α drives NF-κB/iNOS-mediated oxidative stress and blood-brain barrier disruption via MMP-9 upregulation (84, 88). P53-dependent apoptosis is amplified through TNF-α/p38 MAPK crosstalk (86). Secondly, Neuroprotective potential: TNF-α enhances neurotrophin synthesis (BDNF ↑1.9-fold) and suppresses Ca2+ overload-induced excitotoxicity (Choudhary et al., (83)). In neonatal hypoxic-ischemic models, TNF-α pretreatment polarizes microglia toward an anti-inflammatory phenotype (Arg-1+ cells ↑3.5-fold), rescuing neuronal survival (87).

Emerging strategies exploit TNF-α’s duality: transplantation of TNF-α-primed human neural progenitor cells (hNPCs) reduces ischemic lesion volume by 48% and improves motor function in preclinical TBI models, suggesting potential for neurorestorative therapies (87). However, precise temporal modulation—suppressing chronic neurotoxicity while harnessing acute protective signaling—remains critical for clinical translation.

### 3.11 Transforming growth factor-β1

TGF-β1 exists in an intrinsically latent state, requiring activation by the TGF-β-binding protein to form a large inactive complex. Only upon activation can TGF-β1 bind to its receptor and exert biological effects. This cytokine plays a pivotal role in maintaining cellular equilibrium, particularly in regulating neuronal populations, making it inappropriate to categorize as strictly beneficial or detrimental. In the central nervous system, TGF-β1 exhibits dual functions. On one hand, it is widely regarded as an anti-inflammatory cytokine with neuroprotective properties. Post-TBI, elevated TGF-β1 levels synergize with M-CSF and IL-6 to accelerate macrophage polarization, generating reparative macrophage subsets that enhance neuroprotection, angiogenesis, and cell migration (89). *In vitro* TBI models demonstrate TGF-β1’s ability to suppress cortical neuron apoptosis via upregulation of Cav1.2/LTCCs (90). Additionally, TGF-β1 drives microglial transition from neurotoxic to neuroprotective phenotypes within 24 hours post-injury, mitigating axonal dysfunction (Zhao et al., (91)). It also counteracts IL-1β- and TNF-α-induced reactive astrocyte activation, facilitating neural repair (92), and alleviates demyelination and cognitive deficits in rats by modulating ERK1/2 and NF-κB pathways while dampening microglia-mediated inflammation (93).

Conversely, TGF-β1 demonstrates neurotoxic potential by promoting IL-1β/TNF-α production, enhancing apoptosis, and exacerbating oxidative stress via NADPH oxidase-dependent ROS generation (94, 95). Unpublished data from controlled cortical injury models reveal acute-phase TGF-β1 upregulation, which may aggravate neuronal death through MAPK pathway activation and disruption of cortical neuron firing patterns. These paradoxical effects likely stem from model-specific variables (e.g., injury severity, species differences) or temporal variations in post-TBI signaling. For instance, neuroprotection is often observed in subacute phases, whereas neurotoxicity dominates acutely.

Current evidence precludes definitive conclusions about TGF-β1’s net impact in TBI, as its effects hinge on spatiotemporal expression dynamics, microenvironmental concentration gradients, and crosstalk with downstream pathways. Critical barriers to clinical translation include: (1) a lack of longitudinal human studies mapping TGF-β1 fluctuations across TBI phases; (2) unidentified molecular “switches” governing its dual roles; and (3) interspecies disparities in immune microenvironmental responses. Future research must employ multi-omics approaches to resolve TGF-β1’s context-dependent signaling networks and develop microenvironment-responsive modulation strategies before considering therapeutic targeting.

### 3.12 High Mobility Group Box 1 protein

High Mobility Group Box 1 protein (HMGB1) serves dual roles as a nuclear chaperone regulating DNA transcription under physiological conditions and as a damage-associated molecular pattern (DAMP) during cellular injury. Upon cellular damage, extracellular HMGB1 activates Toll-like receptor 2/4 (TLR2/4) and the receptor for advanced glycation end products (RAGE), triggering p38/NF-κB signaling cascades that amplify neuroinflammation (96, 97). This DAMP orchestrates microglial polarization, exacerbating inflammatory cascades in traumatic brain injury (TBI), stroke, and neurodegenerative diseases via the RAGE-NF-κB axis, which induces pro-inflammatory cytokine release and sustains microglial activation (98, 99). Beyond immune modulation, HMGB1 disrupts cerebrovascular integrity by upregulating aquaporin-4, damaging endothelial cells and pericytes, and enhancing blood-brain barrier (BBB) permeability—a mechanism attenuated by glycyrrhizin (GL), a pharmacological HMGB1 inhibitor shown to reduce neuronal HMGB1 translocation, suppress cytokine storms, and improve neurocognitive outcomes in rodent TBI models through RAGE axis blockade (50, 100).

Clinically, HMGB1 demonstrates prognostic relevance: elevated cerebrospinal fluid (CSF) levels in pediatric TBI patients correlate with poor 6-month outcomes, peaking within 72 hours post-injury (101). Mechanistically, HMGB1 exacerbates secondary injury by priming the NLRP3 inflammasome in severe TBI (102) and impairs white matter repair through TLR2/4-mediated inhibition of oligodendrocyte precursor cell proliferation and myelination (103). Despite its promise as a therapeutic target and biomarker, critical knowledge gaps persist regarding isoform-specific effects, temporal dynamics of HMGB1-receptor interactions, and cell-type-specific signaling. For instance, while preclinical studies highlight GL’s efficacy in acute phases, the therapeutic window for HMGB1 inhibition and its impact on long-term recovery remain undefined (104). These uncertainties underscore the need for longitudinal human studies to validate HMGB1’s clinical utility and optimize targeted intervention strategies.

### 3.13 Granulocyte-macrophage colony-stimulating factor

Granulocyte-macrophage colony-stimulating factor (GM-CSF), a pleiotropic cytokine produced by macrophages, T cells, mast cells, endothelial cells, and fibroblasts, functions as a hematopoietic growth factor while exhibiting neuroprotective properties in the central nervous system. In experimental traumatic brain injury (TBI) models, GM-CSF expression remains elevated from 3 to 60 days post-injury, demonstrating a strong correlation with favorable neurological outcomes (105). Mechanistically, GM-CSF attenuates neuroinflammation by suppressing pro-inflammatory cytokine release, curbing reactive microglial proliferation, and expanding regulatory T cell (Treg) populations, thereby reducing lesion volume and accelerating neural repair (106). Recent preclinical evidence highlights its capacity to reverse TBI-induced immunosuppression: in juvenile male rats with TBI-hemorrhage polytrauma, GM-CSF administration fully restored behavioral deficits within 7 days, concurrent with enhanced mitogen responsiveness in splenic and circulatory immune cells and increased astrocyte counts without provoking systemic inflammation (107). These findings collectively suggest that GM-CSF exerts neuroprotection through acute glial activation/proliferation and context-dependent immunomodulation, bolstering endogenous repair mechanisms while maintaining immune homeostasis post-TBI.

### 3.14 Chemokine (C–C motif) ligand 2

The C-C Motif Chemokine Ligand 2 (CCL2)/CCR2 axis plays a pivotal role in neuroinflammation by mediating monocyte recruitment, T lymphocyte differentiation (TH1/TH2 polarization), and natural killer cell activation at injury sites (108). Overexpression of this chemokine-receptor pair exacerbates neuropathology in traumatic brain injury (TBI), as evidenced by elevated serum CCL2 levels in 92 TBI patients, which inversely correlated with favorable outcomes and served as an independent predictor of poor prognosis (109). Preclinical studies demonstrate that CCL2 knockdown or CCR2 antagonism attenuates neuroinflammation by reducing glial activation, suppressing pro-apoptotic gene expression, and improving cognitive recovery in spatial memory tasks (110, 111). However, age-dependent divergences emerge: CCR2 deficiency in pediatric TBI mice paradoxically elevated serum CCL2 without worsening seizure susceptibility or neuroinflammatory responses, while CCR2 antagonism in adult models failed to mitigate tissue damage or epileptogenesis (111). These findings underscore the context-specific nature of CCL2/CCR2 signaling, where therapeutic efficacy may depend on developmental stage, injury chronology, and compensatory pathway activation, necessitating age-stratified approaches for clinical translation.

### 3.15 Glia maturation factor

Glia maturation factor (GMF), predominantly expressed in astrocytes and neurons within the central nervous system, is upregulated in neurodegenerative pathologies such as Parkinson’s and Alzheimer’s disease, with emerging evidence implicating its role in traumatic brain injury (TBI) (112). Mechanistically, GMF drives neuroinflammation through rapid NF-κB pathway activation, as demonstrated in *in vitro* TBI models where GMF overexpression exacerbates oxidative stress-mediated neuronal death by inducing astroglial hyperactivation (113, 114). Conversely, GMF deficiency attenuates neuropathology across experimental models: zebrafish with GMF mutations exhibit suppressed IL-1β/TNF-α expression, reduced radial glial hypertrophy, and diminished acute-phase reactive gliosis post-TBI (115), while GMF-knockout mice show decreased phosphorylated NF-κB, iNOS, and cyclooxygenase-2 levels, alongside mitigated axonal damage and improved motor recovery (236). Critically, GMF ablation reprograms microglia toward an anti-inflammatory phenotype, characterized by progressive declines in TNF-α and IL-6, which correlates with reduced neuronal loss and behavioral deficits (113). Collectively, these findings establish GMF as a master regulator of TBI-associated neuroinflammation, amplifying glial activation, pro-inflammatory cytokine cascades, and oxidative stress during acute injury phases.

### 3.16 Galectin-3

Galectin-3, the predominant member of the β-galactoside-binding lectin family, is a cytoplasmic protein expressed in macrophages, monocytes, and mast cells that orchestrates inflammatory responses, particularly during acute-phase injury (116). Its expression is markedly upregulated in traumatic brain injury (TBI) and spinal cord injury models, with early post-traumatic elevations detected in microglia, cerebrospinal fluid (CSF), and peripheral plasma (117–119). Clinically, plasma Galectin-3 levels correlate positively with Glasgow Coma Scale scores (r = 0.72, p < 0.001), underscoring its potential as a prognostic biomarker for TBI severity (120). Mechanistically, microglia-derived Galectin-3 released into the CSF during acute TBI binds Toll-like receptor 4 (TLR4), driving the expression of pro-inflammatory mediators (IL-1β, IL-6, TNF-α, NOS2) that exacerbate neuronal loss and neuroinflammation (120, 121). Therapeutic interventions targeting this axis—via neutralizing antibodies or genetic knockdown—significantly reduce inflammatory cytokine production and enhance neuroprotection in cortical and hippocampal regions (122, 123). These findings position Galectin-3/TLR4 signaling as a pivotal driver of secondary injury and a promising therapeutic target for mitigating post-TBI neuroinflammation.

### 3.17 Peripheral cytokines in TBI and their clinical values

Following a TBI, tissue and cell damage caused by direct mechanical injury results in the release of numerous damage-associated molecular patterns at the site of injury. This triggers the activation of microglia, astrocytes, and neurons in the brain, which serve as the primary source of acute phase CSF cytokines in TBI. Damage from TBI frequently leads to the breakdown of the blood-brain barrier, allowing DAMPs released into the peripheral blood, inducing systemic inflammation and increasing levels of cytokines in the peripheral blood.

There are few cytokines with clinically significant value in current research reports, with representative examples including IL-6, IL-10, and IL-15. Clinically, many studies support a positive correlation between elevated IL-6 and more detrimental TBI effects (124). Among TBI patients, interleukin-6 (IL-6) was frequently measured and found to have significantly elevated levels in six out of seven studies compared to controls. The elevation was noted as early as six hours after mTBI (125) and persisted for up to six months (35). Similarly, Singhal et al. reported that higher IL-6 levels were related to worse clinical outcomes in a study of patients with TBI (126). In addition, IL-6 correlates to increased risk of elevated cerebral pressure, which can lead to hypoxic areas of the brain due to poor perfusion, herniation, and death (127). In this study, the patients with a higher intracranial pressure (ICP) (≥25 mmHg) had significantly higher IL-6 in the first 17 h after admission. These correlations between elevated IL-6 and poor clinical outcomes may be related to IL-6’s proinflammatory function. If IL-6 is exorbitantly elevated early on, or levels do not fall after acute inflammation, IL-6 may promote detrimental chronic inflammation (128).

In addition, It is important to consider that in cases where the blood-brain barrier remains intact, assessing the levels of IL-6 in CSF or brain parenchyma directly but not the serum level could offer more diagnostic value. In rats, serum IL-6 was significantly increased 90 min after TBI but decreased after 24 h. In contrast, brain tissue IL-6 remained elevated (129, 130). Therefore, relying solely on measurements of peripheral cytokine levels fails to promptly and accurately reflect the dynamic changes of their counterparts within the brain. This may be a key reason why cytokines have proven difficult to use for predicting clinical outcomes in TBI patients.

IL-10 could be considered as another independent marker for TBI prognosis. Elevated serum levels of IL-10 were also associated with mortality (57, 131). In a sample of patients with severe TBI, mortality rates were up to 6 times higher in individuals with higher IL-10 levels (>90 pg/ml) compared to those with lower IL-10 levels (<50 pg/ml) (131). This may attribute to the positive association between elevated levels of IL-10 and GCS severity, BBB dysfunction and mortality in severe TBI (131, 132). Among 166 isolated TBI patients in a clinical study (58), IL-10 emerged as the most prominently affected cytokine in the acute phase following the injury, showing a positive correlation with the severity of the injury. Furthermore, patients who died due to either neurological or non-neurological organ failure exhibited elevated levels of IL-10. However, there are also report indicating that among patients with TBI, the concentration of IL-10 in cerebrospinal fluid was found to be elevated in 26 out of 28 TBI patients, with only 7 of them showing elevated serum IL-10 levels. Taken together, these findings underscore the overall detrimental role of IL-10 in the early stages of TBI, and indicate that IL-10 levels in peripheral blood likely have a potential to become a valuable biomarkers for predicting outcomes specific to brain injury. However, it is questionable whether this prognostic value is universally applicable to all TBI patients. Further studies are required to identify the specific injury stages or patient subtypes where IL-10 is most predictive.

In a Transforming Research and Clinical Knowledge in Traumatic Brain Injury (TRACK-TBI) Pilot Study, IL-15 showed acceptable discriminatory ability for clinical and radiographic TBI, with acceptable AUC values (0.70–0.74) for predicting 3- and 6-month Extended Glasgow Outcome Scale (GOSE) outcomes across TBI severities (133).Yet, the multifunctionality, redundancy, synergy, and antagonism among cytokines limit the reliability of any single peripheral marker as an independent prognostic factor. Current prediction models more commonly incorporate systemic inflammatory indices (e.g., Neutrophil-to-Lymphocyte Ratio [NLR] and Monocyte-to-Lymphocyte Ratio [MLR]) rather than isolated cytokines (134–137). Moreover, intact BBB cases may render serum levels less reflective of CNS inflammation, underscoring a key limitation: peripheral cytokine profiling alone often fails to accurately mirror rapid intraparenchymal changes.

Moreover, predicting long-term disability requires a more nuanced view. Large-scale cohort studies like the TRACK-TBI initiative have been pivotal in demonstrating that the balance between pro- and anti-inflammatory signals is a more powerful prognostic indicator (133). A high IL-6/IL-10 ratio in the acute phase, for instance, is a strong predictor of unfavorable six-month outcomes (GOSE), with an odds ratio of approximately 2.5 for poor recovery, elegantly capturing the concept of an “unresolved” inflammatory state (133). Further validating this, a TRACK-TBI analysis of a broad cytokine panel confirmed that early elevations in pro-inflammatory cytokines were significantly associated with worse outcomes across all TBI severities (133). This prognostic power extends to mild TBI, where specific IL-6, TNF-α, IL-1RA, IL-10, and MCP-1/CCL2 were associated with poor clinical outcome, highlighting the value of measuring inflammatory cytokines in future mTBI research, which still lacks consensus in methodology. Similarly, the Collaborative European NeuroTrauma Effectiveness Research in Traumatic Brain Injury (CENTER-TBI) study has shown that cytokine profiles, including elevated IL-6 and TNF-α, correlate with prognosis in moderate-to-severe TBI, with AUC values around 0.75 for predicting unfavorable GOSE scores at 6 months (45, 138).

In addition, the clinical utility of these cytokines will significantly amplified when integrated with novel blood markers of direct structural brain damage, like Glial Fibrillary Acidic Protein (GFAP) and Ubiquitin Carboxy-Terminal Hydrolase L1 (UCH-L1) (139, 140). Combining structural and inflammatory markers in multi-modal panels, analyzed with machine learning algorithms, will provides superior prognostic accuracy by capturing both the initial impact and the subsequent biological response. However, challenges remain, such as biomarker stability over time and the influence of extracranial injuries, necessitating further validation in diverse populations.

## 4 Cytokines related signaling pathways in TBI

### 4.1 Mitogen-activated protein kinase signaling pathway

The Mitogen-Activated Protein Kinase (MAPK) family, comprising evolutionarily conserved serine-threonine kinases including JNK, ERK1/2, and p38 pathways, transduces extracellular signals to regulate inflammation, apoptosis, and cellular homeostasis. In traumatic brain injury (TBI), the p38/MAPK pathway emerges as a dual mediator—initially driving acute neuroinflammation through microglial activation, yet paradoxically exhibiting neuroprotective potential under specific contexts. Acute-phase p38 activation in microglia promotes sustained pro-inflammatory cytokine release (IL-1β, IL-6, TNF-α) and chronic neuroinflammatory microenvironments, as evidenced by pharmacological interventions: HET0016 inhibition reduces neuronal apoptosis by 62% in immature TBI rats, while curcumin suppresses cytokine upregulation by blocking p38 signaling (141, 142). Conversely, TNF-α-induced p38 activation upregulates KNa1.2 channels, mitigating neuroinflammation and seizure susceptibility in murine TBI models (143). The JNK pathway displays transient neuronal activation post-TBI, with *in vitro* studies implicating its role in mechanical injury-induced apoptosis, though *in vivo* functional validation remains pending (144, 145).

In contrast, ERK1/2 signaling demonstrates persistent neuronal activation, correlating with long-term apoptotic and necroptotic processes even at 30 days post-injury. While MAPK activation may partially sustain baseline neuronal function, therapeutic targeting predominantly focuses on suppressing acute-phase dysregulation. Emerging strategies include intranasal delivery of miRNA-loaded extracellular vesicles to inhibit NLRP3-p38 crosstalk, which attenuates chronic cognitive deficits (146). Notably, FDA-approved MAPK inhibitors for oncology—such as p38 antagonists—show translational potential for TBI, though clinical validation is required to repurpose these agents. This dichotomy underscores the need for temporally precise interventions that balance MAPK’s acute neurotoxic signaling with its context-dependent protective roles.

### 4.2 TLR4/NF-кB

Toll-like receptor 4 (TLR4), a pattern recognition receptor expressed in microglia, astrocytes, and brain-resident macrophages, exacerbates neuroinflammation and white matter damage in traumatic brain injury (TBI) through NF-κB-dependent mechanisms. Upon binding pathogen-associated molecular patterns (e.g., lipopolysaccharide) or damage-associated signals (e.g., IL-1β, TNF-α), TLR4 dimerizes with myeloid differentiation protein 2 (MD-2), triggering canonical NF-κB activation that upregulates pro-inflammatory cytokine synthesis (IL-6, TNF-α, IL-1β) by 3- to 5-fold (147, 148). This signaling axis further impedes neurorepair by blocking astrocyte transition to anti-inflammatory phenotypes during acute TBI, thereby sustaining chronic neuroinflammatory microenvironments (149, 150).

Preclinical evidence underscores the TLR4/NF-κB pathway as a pivotal therapeutic target: curcumin reduces TLR4/NF-κB protein levels in microglia, attenuating neuronal apoptosis and cytokine storms (151). Similarly, hydroxychloroquine, fluoxetine, and omega-3 polyunsaturated fatty acids suppress microglial M1 polarization via this axis, decreasing blood-brain barrier permeability and improving functional recovery (152–154). Pharmacological TLR4 antagonism achieves dual benefits—modulating neuroinflammation through NF-κB inhibition while normalizing autophagic flux, as evidenced by reduced LC3-II/Beclin-1 expression and mitigated hippocampal neuronal loss (155). Despite promising results, clinical translation requires addressing unresolved challenges, including temporal specificity of interventions (acute suppression vs. chronic modulation) and blood-brain barrier penetrance of TLR4 inhibitors. Multitarget approaches combining TLR4/NF-κB blockade with growth factor therapies (e.g., fibroblast growth factors) may optimize neuroprotection while minimizing compensatory inflammatory cascades.

### 4.3 TGF-β1/Smads signaling pathway

The TGF-β1/Smads signaling pathway orchestrates critical post-TBI cellular processes—including proliferation, migration, and immune regulation—through a conserved molecular cascade. Ligand binding initiates TGF-β1 dimerization with type II receptors, recruiting type I receptors to form heterotetrameric complexes that phosphorylate receptor-activated Smads (R-Smads: Smad2/3). These activated R-Smads then complex with Smad4, translocate to the nucleus, and modulate target gene transcription, while inhibitory Smads (I-Smads: Smad6/7) fine-tune signaling intensity (237, 238). Mirroring TGF-β1’s dual roles, this pathway exhibits context-dependent neurotoxicity and neuroprotection. In murine TBI models, TGF-β1/Smad activation exacerbates secondary injury by upregulating NOX1-driven ROS production, amplifying neuroinflammation, and inducing apoptosis (95). Conversely, rat studies demonstrate neuroprotective effects: Smad3 knockdown worsens astrogliosis and neuronal loss (156), while ADAM17-mediated microglial M2 polarization via TGF-β1/Smad signaling reduces neuroinflammation (157). Such diametric outcomes underscore pathway plasticity, where therapeutic low-frequency magnetic stimulation attenuates spinal injury by suppressing Smad2/3 activation, suggesting modality-specific regulation.

Intriguingly, neurotoxic outcomes predominantly emerge in mouse TBI models, whereas neuroprotection is reported in rat studies—a divergence potentially attributable to species-specific Smad isoform expression or microenvironmental variances. This discrepancy highlights the urgency of clarifying TGF-β1/Smad dynamics in human TBI cohorts or iPSC-derived models to establish injury phase-specific roles (acute vs. chronic) and receptor stoichiometry thresholds. Validated human data could guide optimal animal model selection for mechanistic studies, particularly to resolve whether pathway activation exacerbates neuroinflammation in severe TBI or promotes repair in milder cases. Such stratification is essential before leveraging FDA-approved TGF-β modulators (e.g., galunisertib) for TBI therapeutics.

### 4.4 PI3K/Akt/mTOR

The PI3K/AKT/mTOR signaling axis, comprising lipid kinase PI3K isoforms (classes I-III), serine/threonine kinase AKT (AKT1-3), and mTOR complexes 1/2, serves as a master regulator of cellular homeostasis by governing growth, apoptosis, angiogenesis, and glucose metabolism (158, 159). In traumatic brain injury (TBI), secondary injuries involve a complex interplay of neuroinflammation, oxidative stress, apoptosis, and impaired autophagy, driven by key cytokines and their downstream signaling pathways. The PI3K/Akt/mTOR signaling cascade serves as a critical regulator in this context, influencing neuronal proliferation, axon growth, and dendrite formation (160). Upon TBI, extracellular growth factors and tyrosine kinases activate PI3K, leading to Akt phosphorylation and downstream mTOR engagement. While mTOR activation can inhibit autophagy—a process essential for clearing cellular debris—this inhibition may paradoxically hinder nerve repair in certain scenarios (1). However, recent studies demonstrate a predominant protective role: activation of PI3K/Akt/mTOR reduces neuronal apoptosis, attenuates inflammation, and improves cognitive and sensorimotor functions post-TBI (160–162). For instance, interventions like ursolic acid and fisetin enhance this pathway to suppress ferroptosis and oxidative stress, yielding net neuroprotective effects despite autophagy suppression (160, 162). Dual effects are evident, as overactivation might contribute to maladaptive responses, but targeted modulation (e.g., via miR-3571 or 4,4’-dimethoxychalcone) consistently promotes recovery in animal models (163, 164).

Akt also interacts with GSK-3β, a pro-apoptotic mediator that induces mitochondrial permeability transition and apoptosis primarily in neurons and microglia (161, 165). In cortical neurons, GSK-3β phosphorylation post-TBI inhibits survival signals and promotes apoptosis via transcription factor modulation (165). In microglia, GSK-3β exacerbates inflammation by phosphorylating PTEN, suppressing PI3K/Akt, and driving pro-inflammatory polarization, leading to elevated TNF-α and IL-6 levels (161, 166). Akt counteracts this by phosphorylating GSK-3β at Ser9, inhibiting its activity and reducing apoptosis and inflammation in these cell types (161, 167).

**Figure 4:**
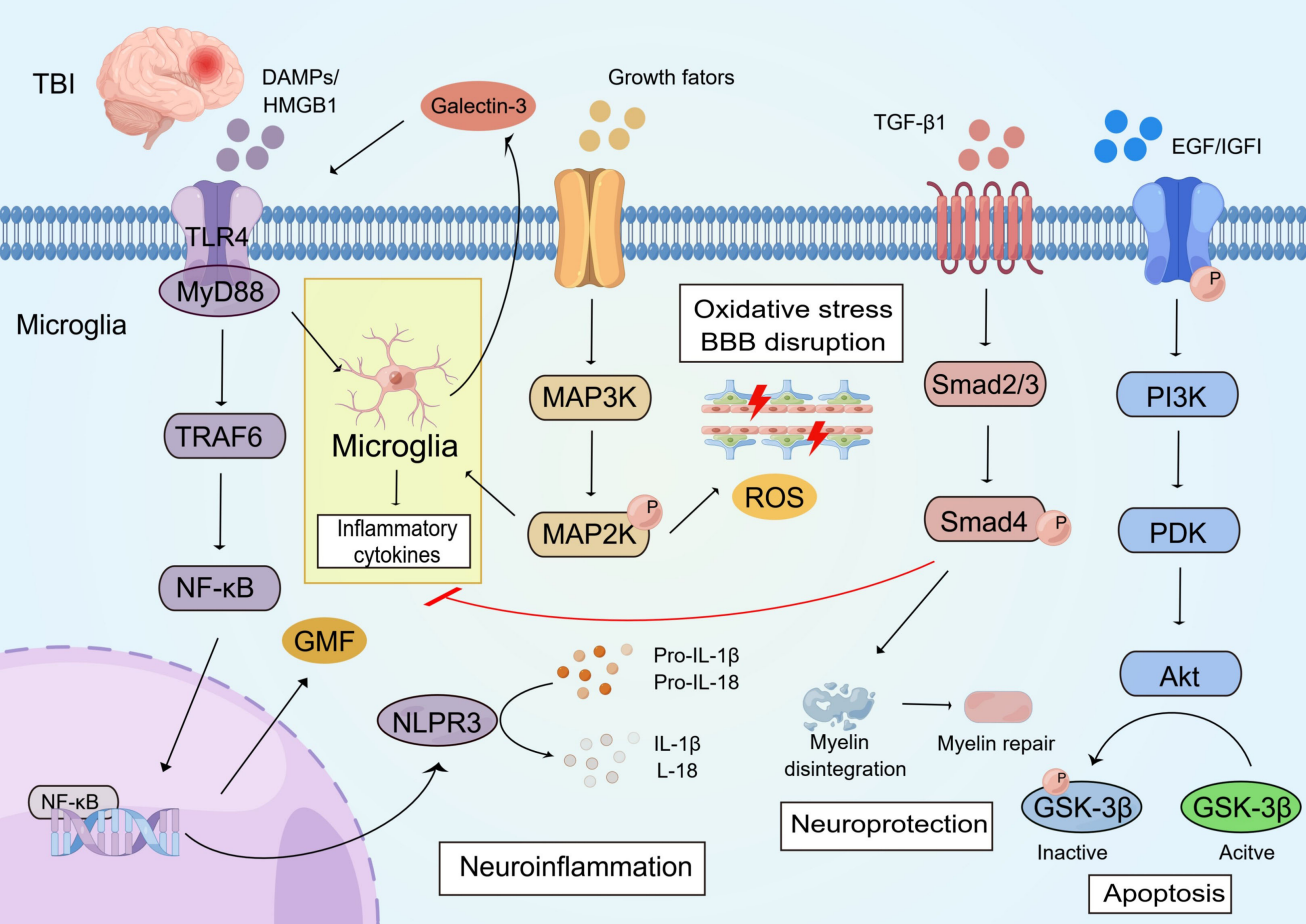
An overview of cytokine factors related signaling pathways that are involved in TBI. I. Injured neurons release damage-associated molecular patterns (DAMPs) and HMGB1, which bind to TLR4. This activates microglia to produce more inflammatory factors and Galectin-3. At the same time, it initiates a cascade reaction with MyD88, promoting the entry of NF-κB into the nucleus to bind to the promoter and up-regulate the expression of NLPR3 and GMF. This accelerates the release of IL-1β and IL-18. Galectin-3 can also initiate an inflammatory response by binding to the TLR4 receptor. II. The binding of ligands (growth factors and cytokines) to specific receptors triggers a cascade reaction of MAPKs. These are activated by phosphorylation and ultimately induce microglia activation, oxidative stress, and blood-brain barrier damage. III. TGFβ1 binds to the receptor and initiates Smads protein phosphorylation. This inhibits microglia activation and protects the nerves, preventing myelin shedding. IV. After TBI, epidermal growth factor and insulin growth factor bind to tyrosine kinase receptors, initiating a cascade reaction that activates Akt. Akt participates in apoptosis by regulating the phosphorylation of the pro-apoptotic mediator GSK-3β.

In summary, the cytokine-driven secondary injury cascade in TBI involves interconnected signaling pathways (**Figure 4**). Pro-inflammatory cascades predominate acutely: injured neurons release DAMPs and HMGB1, which engage TLR4 on microglia, triggering MyD88-dependent NF-κB nuclear translocation to upregulate NLRP3 and GMF expression, thereby accelerating IL-1β and IL-18 release; Galectin-3 amplifies this response via TLR4 binding, while ligand-induced MAPK (p38/JNK/ERK) phosphorylation promotes microglial activation, oxidative stress, and blood-brain barrier disruption—collectively driving early apoptosis, edema, and gliosis. In contrast, resolving pathways exert counter-regulatory effects: TGF-β1 binding to its receptor initiates Smad phosphorylation, inhibiting microglial activation, preserving neuronal integrity, and preventing myelin shedding, thus supporting neuroprotection and repair. Similarly, growth factor (EGF/IGF)-mediated activation of PI3K/Akt modulates apoptosis through GSK-3β phosphorylation, offering context-dependent balancing of inflammation, autophagy, and survival signaling. Sustained dysregulation—prolonged pro-inflammatory dominance with inadequate resolving input—fuels chronic neurotoxicity, neurodegeneration, and long-term sequelae. This integrated pathway crosstalk highlights the therapeutic promise of phase-specific, multimodal interventions that enhance protective arms (e.g., TGF-β/Smad or Akt activation) while suppressing maladaptive pro-inflammatory loops, accounting for injury severity and translational challenges observed in preclinical and clinical studies.

### 4.5 Signaling pathway crosstalk: the reality behind therapeutic failures

**Figure 5:**
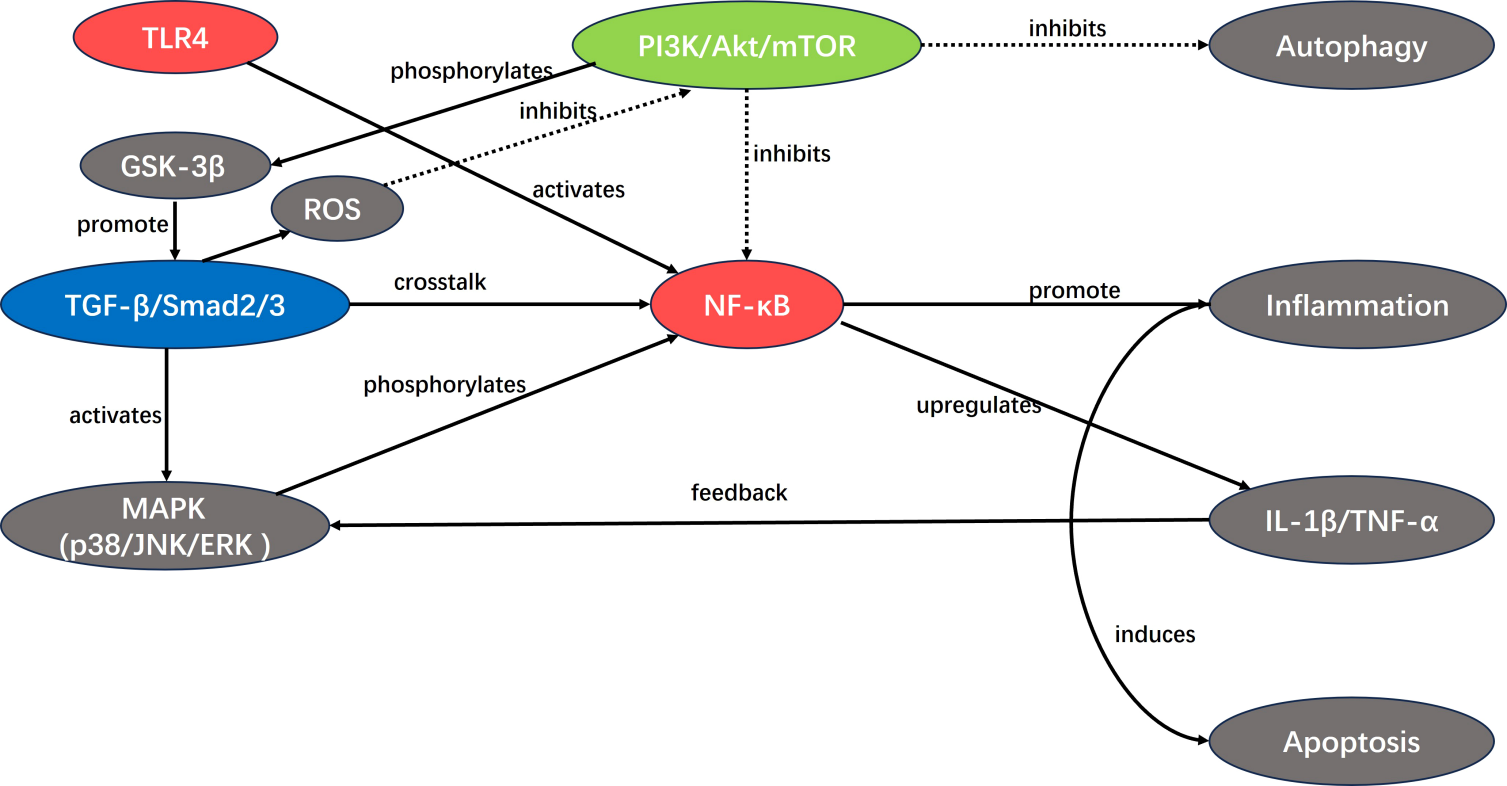
Key signaling pathways and crosstalk in traumatic brain injury. This directed network diagram illustrates the major signaling pathways involved in TBI neuroinflammation, including TLR4/NF-κB (upstream activator of inflammation), MAPK (p38, JNK, ERK1/2; parallel regulator of apoptosis and autophagy), TGF-β/Smad (modulator of gliosis and repair with dual roles), and PI3K/Akt/mTOR (regulator of autophagy and apoptosis, interacting with GSK-3β). Nodes represent key pathway components, cytokines (e.g., IL-1β/TNF-α as inputs), and outcomes (e.g., Inflammation, Apoptosis, Autophagy). Solid arrows indicate activation or positive regulation, dashed arrows denote inhibition or negative feedback. Crosstalk is highlighted: TLR4 activates NF-κB, leading to IL-1β/TNF-α release, which feeds back to MAPK (p38/JNK) forming a positive loop; NF-κB upregulates inflammation but is inhibited by Akt (negative feedback from PI3K/Akt/mTOR); TGF-β/Smad activates NF-κB (pro-inflammation) or inhibits MAPK (repair), showing duality; PI3K/Akt/mTOR phosphorylates GSK-3β to indirectly regulate Smad transcription, while TGF-β feeds back to inhibit PI3K via ROS. NF-κB serves as the central hub converging all pathways, leading to inflammation/apoptosis outputs.

While this review has discussed the roles of key signaling pathways such as MAPK, TGF-β, PI3K/Akt, and TLR4/NF-κB, it is a vast oversimplification to view them as independent, linear cascades. A more accurate depiction is of a highly integrated and redundant signaling network, where extensive crosstalk between pathways is the norm, not the exception (168). The failure to appreciate this complexity is a fundamental reason why numerous therapeutic agents targeting these pathways have failed in clinical trials for traumatic brain injury (TBI) (169). Molecules like NF-κB and MAPKs do not act in isolation but serve as critical signaling integration nodes, receiving inputs from multiple upstream pathways and coordinating a complex downstream response (**Figure 5**).

The MAPK-NF-κB axis provides a prime example of this intricate crosstalk. MAPK subfamilies (p38, JNK, ERK) can directly phosphorylate and activate the upstream IKK complex, a key step in NF-κB activation (170). Conversely, NF-κB can, in turn, regulate the transcription of genes involved in the MAPK pathway, creating complex feedback or feed-forward loops that sustain inflammation. This crosstalk leads to context-dependent and sometimes paradoxical pathological outcomes in TBI. For instance, in the immature brain, TBI-induced neuroinflammation and pyroptosis are predominantly driven by a p38 MAPK → NF-κB → NLRP3 inflammasome signaling cascade (141). In this scenario, therapeutic strategies solely targeting the TLR4 pathway might be ineffective, as the primary driver of NF-κB activation is MAPK signaling. In contrast, in many adult TBI models, the TLR4/MyD88 axis is a major upstream activator of NF-κB (171). This age- and context-dependent activation pattern is a direct manifestation of signaling network complexity.

This inherent complexity provides a rational explanation for the failure of single-target drugs. The principles of signaling redundancy and compensatory activation mean that inhibiting a single node (e.g., a specific kinase) can be futile, as the signal flow can simply be rerouted through alternative, parallel pathways to reactivate the same downstream effectors (169). Therefore, future therapeutic development for TBI must evolve beyond the “one-target, one-drug” paradigm (172). Two promising strategies are emerging: a) Multi-target Combination Therapy: This approach involves simultaneously targeting multiple critical nodes within the network to achieve a synergistic effect and prevent compensatory signaling. For example, combining a p38 MAPK inhibitor with a TLR4 antagonist could provide more comprehensive suppression of neuroinflammation than either agent alone. b) Targeting Upstream Master Switches: Instead of targeting individual downstream pathways, a more effective strategy may be to inhibit upstream “master switches” that control the entire inflammatory network. The inflammasome represents such a target. As a central platform for the activation of caspase-1 and the maturation of potent cytokines like IL-1β and IL-18, inhibiting inflammasome assembly or function could simultaneously block inputs from multiple upstream pathways (TLR4, MAPK, etc.), offering a more robust anti-inflammatory effect (173, 174).

In conclusion, understanding and targeting the intricate crosstalk between signaling pathways, rather than the pathways themselves, is paramount. This shift in perspective is essential for overcoming past therapeutic failures and designing the next generation of effective neuroprotective treatments for TBI.

### 4.6 Clinical utility and future of cytokine associated pathway in TBI

The signaling pathways themselves are becoming direct clinical targets in TBI. The inflammasome pathway, particularly NLRP3, has emerged as a high-priority target, supported by human evidence showing that genetic variations in the NLRP3 gene are associated with outcomes after TBI. This has spurred clinical trials for drugs that modulate this pathway, such as glibenclamide, which has been evaluated in clinical trials for its potential to improve motor and neuropathological outcomes in CCI patients, demonstrating reduced contusion expansion and improved functional scores in Phase II studies (175). The STAT3 pathway, a key downstream effector of IL-23, also has clear clinical relevance, with human TBI tissue showing robust and sustained IL-23R/STAT3 activation (176). Finally, the TLR4/NF-κB pathway remains a critical focus, as circulating levels of its ligands, like HMGB1, are elevated in TBI models and correlate with poor outcomes. This has led to clinical investigation of repurposed drugs like atorvastatin, which has pleiotropic anti-inflammatory effects including TLR4 modulation, with meta-analyses suggesting a potential for improved neurological outcomes in moderate-severe traumatic brain injury, including reduced recurrence rates and better GCS scores (177, 178) while its potential benefit in critically ill adult patients is uncertain. Tighter integration of these pathways with cytokine modulation—such as NLRP3 inhibitors reducing IL-1β release—could directly mitigate TBI outcomes by curbing the cytokine storm.

The true frontier, however, lies in using these insights for personalized medicine. The future requires dynamic monitoring of both cytokine and pathway activity to identify patient-specific “inflammatory endotypes”. Inflammatory endotypes refer to distinct patient subgroups in traumatic brain injury (TBI) identified by unique patterns of systemic or neuroinflammatory responses (e.g., differential cytokine profiles such as elevated IL-6-driven clusters), which reflect shared underlying pathobiological mechanisms and are associated with varying clinical outcomes and treatment responses (138, 179). This would allow for the implementation of chronotherapeutic strategies—for instance, targeting a patient with an unresolved inflammatory profile with specific immunomodulatory therapy in the subacute phase. The development of novel technologies like rapid, bedside multiplex biosensors will be instrumental in making this vision a reality, ultimately allowing us to deliver the right treatment, to the right patient, at the right time.

## 5 Main immune cells involved in TBI

### 5.1 Neurons

Historically viewed as passive victims of the inflammatory storm, a paradigm shift now positions neurons as active and integral participants in the neuroimmune response following TBI (40, 180). They are not merely targets but also initiators, producers, and modulators within this complex network, fundamentally shaping the course of secondary injury and recovery (180).

As initiators of inflammation, injured neurons are a primary source of Damage-Associated Molecular Patterns (DAMPs). Due to their high metabolic rate and mitochondrial abundance, damaged neurons release significant amounts of DAMPs like mitochondrial DNA and ATP, which are potent activators of pattern recognition receptors on microglia and astrocytes, thereby igniting the initial inflammatory cascade (181). Furthermore, neurons are direct and rapid producers of cytokines (40, 180). Mechanical trauma alone is sufficient to trigger a robust transcriptional and translational response in neurons (182, 183). Our own work has demonstrated that cultured cortical neurons subjected to mechanical stretch injury rapidly release a broad spectrum of cytokines and chemokines, including IL-1β, IL-6, TNF-α, and even the anti-inflammatory cytokine IL-10 (182). This neuron-autonomous response represents a critical first wave of immune signaling, occurring even before significant infiltration of peripheral immune cells.

Perhaps most critically, neurons are also primary targets and modulators of the cytokine milieu, creating intricate feedback loops that directly impact neural circuit function (180, 184). Cytokines released by glia and other neurons can act as “atypical neuromodulators,” fundamentally altering neuronal excitability and synaptic transmission (185). For instance, TNF-α and IL-6 have been shown to directly modulate the biophysical properties of voltage-gated sodium channels, leading to aberrant firing patterns in uninjured neurons that contribute to network hyperexcitability and post-traumatic epilepsy (186, 187).

Finally, the role of neuron-derived signals demonstrates the principle of temporal duality—the time-dependent functional switch whereby the same mediator exerts protective effects acutely but shifts to maladaptive, neurotoxic roles when chronically sustained (22, 23, 188). Transforming growth factor-beta 1 (TGF-β1), classically considered neuroprotective, can be secreted by neurons in the chronic phase post-TBI. In this context, emerging evidence suggests it can switch its function to promote neuronal apoptosis and necroptosis through sustained activation of pathways like ERK1/2, thus contributing to long-term neurodegeneration (189). In essence, neurons not only sense and respond to the immune environment but actively shape it, with their signals capable of both adaptive and maladaptive functions over time (180, 184). Understanding this dynamic interplay is a key frontier in developing therapies that can restore healthy neuron-immune communication.”

### 5.2 Microglia

Microglia serve as key sentinels in maintaining homeostasis and health within the central nervous system. Upon activation, microglia rapidly release both anti-inflammatory and pro-inflammatory factors, which further modulate peripheral immune cells and resident CNS populations to either induce or impede inflammation through pathways such as TLR4/NF-κB and NLRP3 inflammasome (as detailed in the “Cytokines related signaling pathways in TBI” section). Microglia exhibit a distinct property—plasticity—enabling them to respond differentially in various environments, altering their phenotype and function to adapt to changes (13, 190). The abnormal physiological state of microglia dynamically shifts depending on the microenvironment and may manifest as neurotoxic or neuroprotective effects. An intriguing phenomenon in TBI is the biphasic pattern of microglial proliferation: their numbers peak around the seventh day post-injury, then decline, with a secondary small peak reappearing at one month, persisting even up to a year (12, 191, 192). As one of the first responders after TBI, microglia detect cellular debris and inflammatory mediators released from damaged cells, remodeling their morphology via MAPK signaling activation (193). They retract branches and expand their cytosol to adopt an amoeboid form, facilitating enhanced phagocytosis of debris while potentially mitigating further nervous system damage (20, 193). As a key mediator of neuroinflammation, sustained microglial activation—often perpetuated by PI3K/Akt/mTOR dysregulation—leads to adverse outcomes in later injury stages, hindering repair processes. Microglia promote persistent neuropathology and long-term functional impairments in neuronal homeostasis by sustaining cytokine storms and oxidative stress (13, 194, 195). Thus, strategies targeting microglial senescence and depletion hold potential to ameliorate neuroinflammation via modulation of JAK/STAT and NF-κB pathways (196). Studies have verified that pre-TBI microglial reduction favors neural repair: depletion reverses long-term brain tissue loss, improves neuronal maintenance, and fosters synapse recovery by attenuating pro-inflammatory polarization (12, 194, 195).

### 5.3 Astrocytes

Astrocytes are critical for CNS development and, like microglia, exhibit remarkable plasticity in response to TBI. Neurotoxic astrocytes amplify pro-inflammatory responses by upregulating cytokines such as IFN-γ, TGF-β, and IL-1α, primarily through NF-κB and JAK/STAT signaling pathways (as detailed in the “Cytokines related signaling pathways in TBI” section), contributing to neuroinflammation and neuronal damage (20, 197). In contrast, neuroprotective astrocytes promote anti-inflammatory responses, releasing brain-derived neurotrophic factor (BDNF) and vascular endothelial growth factor (VEGF) to support repair and synaptic homeostasis (13, 20). Upon TBI-induced stimulation, astrocytes undergo gliosis, characterized by hypertrophy and a multibranched morphology, which can progress to glial scarring, impeding neural repair (198, 199). Unlike microglia, astrocytes typically remain localized at the injury site, swelling and sustaining activation, leading to persistent glial scars that may last for months (133, 200). Beyond scarring, astrocytes modulate the inflammatory milieu by releasing both pro-inflammatory (e.g., IL-1β, TNF-α) and anti-inflammatory (e.g., IL-10) factors, influencing synaptic function and potentially promoting neuronal hyperexcitability, which contributes to post-traumatic epilepsy (20, 199). Modulating astrocyte phenotypes within specific temporal windows—suppressing neurotoxic astrocytes via targeted inhibition of NF-κB or JAK/STAT pathways while enhancing neuroprotective astrocytes—offers a promising strategy to mitigate chronic inflammation and improve long-term TBI outcomes (Obukohwo et al., (13)).

### 5.4 Neutrophil

Neutrophils, the most abundant white blood cells, maintain a dynamic balance in the bloodstream under normal physiological conditions (201). Following TBI, neutrophils are rapidly recruited by chemokines to the injury site, becoming the first responders in neuroinflammation. In a controlled cortical impact (CCI) mouse model, neutrophil infiltration was detected in damaged brain tissue within one hour post-injury (202). A clinical postmortem study of 305 TBI cases reported neutrophil infiltration in 43% of samples as early as 5 minutes post-injury, highlighting their swift response (21). DAMPs released during TBI activate neutrophils through cytokine-regulated signaling pathways, such as TLR4/NF-κB (triggered by HMGB1), leading to the release of pro-inflammatory and chemotactic factors that amplify local inflammation (12, 21). For instance, cytokines like IL-6 and TNF-α activate the STING pathway, promoting oxidative stress and neuronal death, while IL-8 and GM-CSF enhance neutrophil survival and inflammation via JAK/STAT signaling (17, 21). Neutrophils also exhibit phagocytic activity, clearing damaged tissue and cellular debris at the injury site. A key mechanism exacerbating secondary injury is the formation of neutrophil extracellular traps (NETs), induced by cytokines such as IL-1β through IRE1α/ASK1/JNK and FOXO1-VCAN pathways, which disrupt the blood-brain barrier (BBB) and intensify neuroinflammation (17, 21). Moreover, neutrophils release cytotoxic mediators like inducible nitric oxide synthase (iNOS) and myeloperoxidase (MPO), further damaging tissues and the BBB (21). Targeting these pathways offers therapeutic promise: for example, Cl-amidine inhibits NET formation, reducing neuroinflammation, neuronal apoptosis, and neurological deficits via the STING-dependent IRE1α/ASK1/JNK pathway (202). Overall, modulating neutrophil responses within specific temporal windows—focusing on cytokine-driven pathways like TLR4/NF-κB and JAK/STAT—could mitigate chronic inflammation and improve TBI outcomes (12).

### 5.5 B cell

B cells, key leukocytes in adaptive immunity, produce high-affinity antibodies and secrete cytokines, yet their role in TBI remains underexplored, with limited clinical analyses of B cell populations. In a study of 20 severe TBI (sTBI) cases, peripheral CD5+/CD19+ B cells showed no significant differences from controls (203). However, recent preclinical evidence indicates B cells aid injury repair. In the TBI microenvironment, nascent B cells are activated via the Toll-like receptor (TLR)-MyD88-dependent signaling pathway (as detailed in the “Cytokines related signaling pathways in TBI” section), producing cytokines that modulate inflammation and accelerate recovery (204). In a mouse CCI model, exogenous B cells enhanced neuroprotective IL-10 and TGF-β production in later stages, exerting sustained anti-inflammatory effects and fostering a neuroprotective niche through reciprocal interactions with infiltrating peripheral myeloid cells via JAK/STAT and NF-κB pathways (205, 206). Additionally, intraperitoneal B cell injection post-CCI reduced microglial activation and ameliorated late-stage brain injury (239). These findings suggest B cells contribute to TBI secondary injury resolution through cytokine-regulated signaling, warranting further investigation into their therapeutic potential.

### 5.6 Tregs

Regulatory T cells (Tregs), a subset of CD4+ T lymphocytes, are renowned for their anti-inflammatory properties and crucial role in immune homeostasis, modulating responses through CTLA4 upregulation and immunosuppressive cytokines like IL-10 and TGF-β. In the healthy brain, Tregs are scarce (44). Post-TBI, circulating Tregs increase during the acute phase, correlating with favorable outcomes (207, 208). Murine TBI models show elevated Treg levels in peripheral blood and brain tissue during subacute injury, with high chronic-phase Tregs in repetitive mild TBI (rmTBI) (209, 210). Tregs partially mediate IL-33/ST2 signaling neuroprotection, reducing lesion size and deficits; CD25 antibody-mediated Treg depletion abolishes IL-33 benefits, underscoring their protective involvement via FOXP3 and JAK/STAT pathways (as detailed in the “Cytokines related signaling pathways in TBI” section) (209, 211). Chronically, Tregs mitigate astrocyte activation and release trophic factors like IL-10 to promote regeneration (212). However, acute Treg depletion reduces T-cell infiltration, astrocytosis, IFN-γ expression, and motor deficits, suggesting potential acute detriment (212). In summary, Tregs exert neuroprotection chronically through cytokine-regulated pathways TNF-α/NF-κB, yet their acute role remains debated, warranting further cytokine-specific modulation studies (208).

## 6 TBI-related neurological disorders

Traumatic brain injury (TBI) initiates heterogeneous secondary injury cascades comprising excitotoxicity, oxidative stress, mitochondrial dysfunction, axonal degeneration, neuroinflammation, and regulated cell death mechanisms, which collectively drive progressive neurological deterioration (213). This review examines cytokine-mediated mechanisms underlying these pathological processes, emphasizing their time-dependent duality. While acute-phase cytokine signaling orchestrates essential neuroprotective immune surveillance to mitigate initial damage, dysregulated chronic inflammation disrupts immune homeostasis, fostering neurodegenerative sequelae including epilepsy, Alzheimer’s disease, and chronic traumatic encephalopathy (214, 215). Critically, the transition from reparative to maladaptive chronic-phase inflammation hinges on cytokine network imbalances, receptor desensitization thresholds, and feedforward loops between innate immune cells and damaged parenchyma, necessitating temporally precise therapeutic interventions.

### 6.1 Chronic neuroinflammation

**Figure 6:**
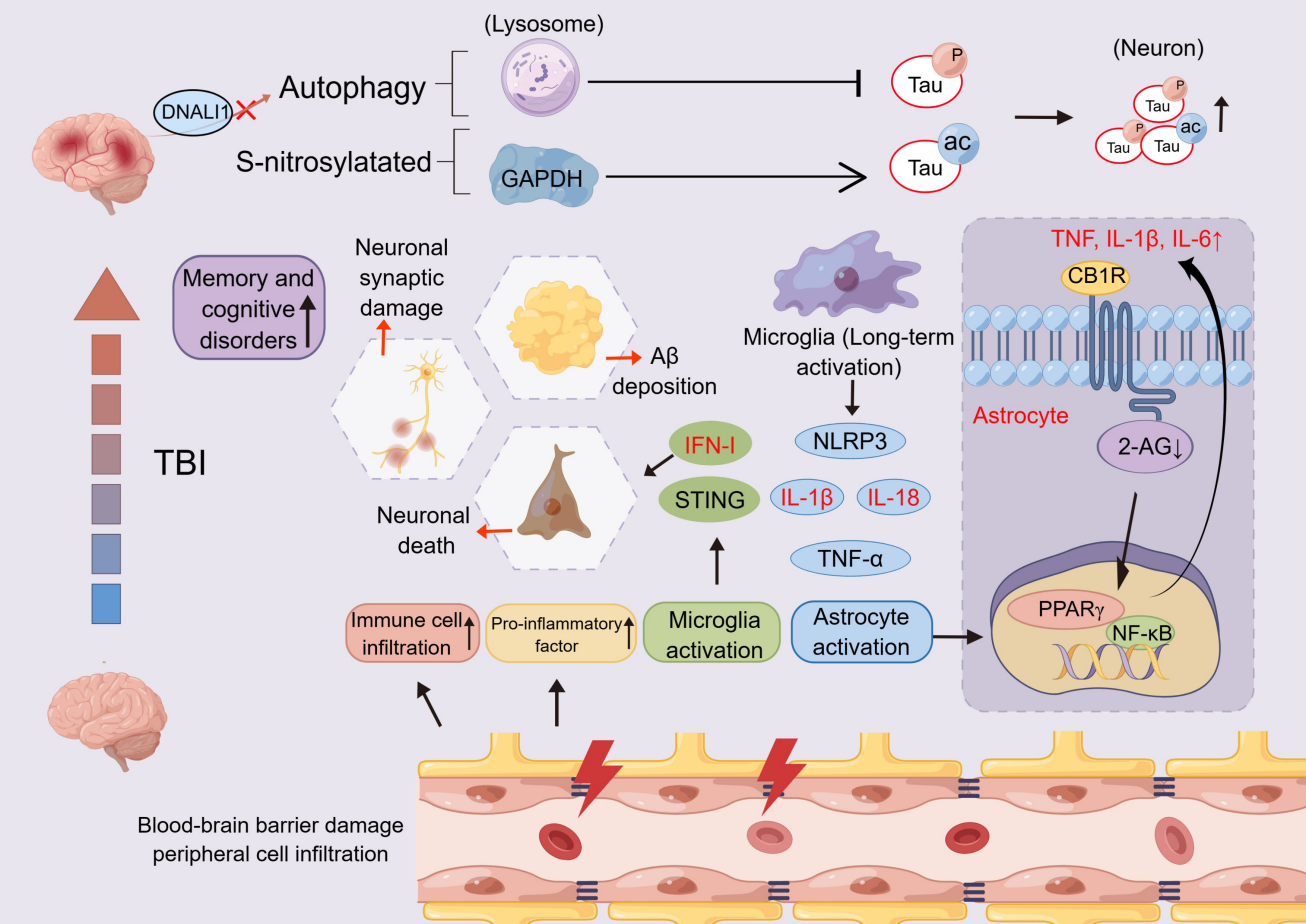
Pathogenesis of neurodegeneration caused by TBI. Secondary injury in TBI initiates neuropathic processes via intricate mechanisms. Post-TBI, the disruption of the blood-brain barrier allows for the infiltration of peripheral immune cells, leading to the activation of microglia and astrocytes. In the acute neuroinflammatory phase, the IFN-I-dependent activation of the STING signaling pathway contributes to neuronal apoptosis. Subsequently, chronic microglial activation triggers the NLRP3 inflammasome, resulting in the production of interleukin-1β and interleukin-18. The ongoing presence of inflammatory factors perpetuates the disruption of blood-brain barrier integrity and neuronal synapses, while also triggering the activation of astrocytes. Traumatic brain injury results in the reduction of astrocyte 2-arachidonoylglycerol (2-AG) levels, activation of the cannabinoid receptor type 1 (CB1R)-PPARγ signaling pathway, and increased expression of pro-inflammatory factors such as TNF, IL-1β, and IL-6, thereby worsening the progression of chronic neuroinflammatory disease. Persistent inflammation following TBI can hinder the clearance of p-Tau and Aβ due to DNALI1 inhibition of autophagic vesicle and lysosome binding, ultimately impeding autophagic flow. Additionally, S-nitrosylation of GAPDH can elevate neuronal ac-tau levels, resulting in mislocalization and subsequent neurodegeneration. The abnormal accumulation of p-Tau, Aβ, and ac-tau serves as a pathological indicator of Alzheimer’s disease and dementia.

The acute-to-chronic neuroinflammatory transition following traumatic brain injury (TBI) is driven by persistent microglial activation, wherein acute-phase IFN-I/STING signaling dysregulation primes chronic maladaptive responses (78). Sustained NLRP3 inflammasome assembly with caspase-1 facilitates chronic microglial hyperactivity, perpetuating IL-1β/IL-18 release and neuroinflammatory cascades that induce long-term neurobehavioral deficits (216). Experimental models of repetitive low-level blast (rLLB) exposure recapitulate this progression: mice exhibit persistent microglial NLRP3 activation concurrent with impaired spatial memory and anxiety-like behaviors across acute and chronic phases (217) (**Figure 6**). Mechanistically, chronic IL-1β overexpression disrupts blood-brain barrier integrity, amplifies perivascular macrophage recruitment, and suppresses synaptic plasticity markers (217). Therapeutic targeting of this axis demonstrates clinical potential—IL-1 receptor knockdown in closed-head injury models attenuates astrogliosis while selectively upregulating neuroprotective chemokines, conferring sustained neuroprotection and functional recovery (218). These findings position IL-1β signaling as both a biomarker of chronic inflammation and a tractable target for interrupting TBI-induced neuroimmune dysregulation.

### 6.2 Chronic traumatic encephalopathy

Chronic traumatic encephalopathy (CTE), a progressive tauopathy characterized by perivascular hyperphosphorylated tau (p-tau) neurofibrillary tangles within sulcal depths, manifests clinically as mood dysregulation, cognitive-motor decline, and memory impairment (219). Central to its pathogenesis is the dissociation of microtubule-stabilizing tau proteins following TBI-induced hyperphosphorylation—a process enabling tau oligomerization, dendritic mislocalization, and subsequent neurofibrillary tangle formation (220, 221). Emerging evidence implicates TBI-mediated autophagic dysfunction in CTE progression: axonemal dynein intermediate chain DNALI1 obstructs autophagosome-lysosome fusion post-injury, impairing p-tau clearance and driving a threefold increase in tau oligomerization (222) (**Figure 6**). This autophagic blockade creates a self-perpetuating cycle wherein accumulated p-tau further disrupts microtubule integrity, synaptic vesicle transport, and neuronal homeostasis, establishing CTE’s pathognomonic neuropathological and clinical trajectories.

### 6.3 Alzheimer’s disease

Epidemiological and molecular evidence increasingly implicates traumatic brain injury (TBI) as a risk amplifier for Alzheimer’s disease (AD), with population studies demonstrating a dose-dependent relationship where severe TBI elevates AD diagnosis rates compared to mild cases (6). This association is mechanistically underpinned by TBI-induced pathological convergence on AD hallmarks: chronic neuroinflammation perpetuates blood-brain barrier dysfunction and tauopathy, while TBI-triggered neuronal tau acetylation (ac-tau) mimics AD-specific post-translational modifications that drive microtubule destabilization, axonal transport failure, and neurofibrillary tangle formation (7, 223) (**Figure 6**). Murine models reveal multifactorial pathogenesis—astrocytic dysregulation of 2-arachidonoylglycerol (2-AG) metabolism via the CB1R-PPARγ axis exacerbates neuroinflammation and potentiates phosphorylated tau accumulation, while impaired ubiquitin-proteasome function delays clearance of Aβ oligomers and tau aggregates (224, 225). These cascades collectively establish a self-reinforcing loop where synaptic damage and chronic traumatic encephalopathy (CTE)-like pathology accelerate AD progression. Despite progress, critical knowledge gaps persist regarding temporal windows for therapeutic intervention and the relative contributions of amyloidogenic versus non-amyloidogenic pathways in TBI-AD comorbidity.

### 6.4 Post-traumatic epilepsy

Post-traumatic epilepsy (PTE), affecting up to 33% of severe TBI patients, arises from a neuroinflammatory milieu where glial dysregulation and immune cell infiltration disrupt neural homeostasis (226). Astrocytes—constituting 90% of human brain cells—ordinarily regulate extracellular potassium and glutamate balance, but TBI-induced activation transforms them into pathological actors that elevate seizure susceptibility through ionic disequilibrium and excitatory circuit remodeling (227, 228). Concurrently, cytokine storms driven by IL-1β/IL-1R signaling exacerbate epileptogenesis: clinical studies reveal that elevated CSF/serum IL-1β ratios and the rs1143634 SNP variant increase PTE risk, while preclinical models demonstrate IL-1β-mediated prolongation of ictal discharges via Ca2+/GABAergic pathway modulation (229, 230). This cytokine further amplifies neurotoxicity by inducing astrogliosis, blood-brain barrier breakdown, and peripheral leukocyte infiltration—pathological hallmarks of epileptic foci (231, 232).

Complementing IL-1β’s role, the HMGB1/TLR4 axis emerges as a dual-phase regulator of TBI-induced epileptogenesis. Released during acute injury, HMGB1 activates neuronal TLR4 receptors in hippocampal circuits, lowering seizure thresholds and potentiating chronic temporal lobe epilepsy resistant to conventional anticonvulsants (233, 234). Preclinical evidence indicates HMGB1-TLR4 blockade reduces seizure frequency in chronic PTE models, likely through NF-κB pathway inhibition and glutamate excitotoxicity mitigation. These findings collectively position neuroinflammatory signaling as both biomarker and therapeutic target—though clinical translation requires resolving critical knowledge gaps, including temporal windows for cytokine modulation and combinatorial targeting strategies to address pathway redundancies.

## 7 Summary

Post-TBI neuroinflammation drives secondary neurodegeneration through spatiotemporally dynamic cytokine networks (e.g., IL-1β, TNF-α, TGF-β1, HMGB1, Galectin-3) that converge on canonical signaling hubs such as TLR4/NF-κB, MAPK, TGF-β/Smads, and PI3K/Akt/mTOR, orchestrating maladaptive plasticity across acute (<24h), subacute (1-7d), and chronic (>7d) phases. A central theme is the temporal duality of these pathways: acute Galectin-3/TLR4 signaling amplifies microglial IL-1β/IL-6 release via NF-κB for initial debris clearance and protection, while chronic persistence exacerbates neurodegeneration in rodent models, though TGF-β1/Smad3 exhibits context-dependent neuroprotection in murine studies by modulating gliosis and repair. Concurrently, MAPK (p38/JNK/ERK) and PI3K/Akt/mTOR govern the autophagic-apoptotic balance, where acute mTOR inhibition enhances neuroprotective autophagy to mitigate neuronal loss, yet risks synaptic disruption if prolonged, as seen in preclinical models of ferroptosis and oxidative stress. These cascades induce region-specific dysfunction—hippocampal HMGB1/TLR4-mediated glutamate excitotoxicity lowers seizure thresholds, while frontal IL-1β/NF-κB impairs GABAergic inhibition—manifesting as post-traumatic epilepsy (up to 33% incidence), CTE-like tauopathy, and affective-cognitive deficits. FDA-approved modulators (e.g., p38/MAPK inhibitors, mTOR antagonists) demonstrate preclinical efficacy in normalizing neuroimmune crosstalk, but clinical translation demands addressing temporal therapeutic windows and pathway redundancy in human TBI subtypes to harness acute protection while curbing chronic neurotoxicity.

## 8 Future directions

Current research on TBI neuroinflammation reveals a complex landscape where cytokine networks exhibit temporal duality, driving acute protection but chronic pathology through interconnected signaling pathways like TLR4/NF-κB and PI3K/Akt/mTOR, as highlighted in recent reviews. However, persistent challenges include the limited translation of preclinical findings to clinical settings, owing to pathway redundancy, species-specific differences, and undefined therapeutic windows that hinder effective modulation of inflammatory cascades. To overcome these limitations, future studies should prioritize multi-omic integration to map cytokine dynamics at single-cell resolution, enabling identification of phase-specific biomarkers for patient stratification and personalized interventions. Additionally, advancing from isolated pathway targeting to combinatorial strategies could address crosstalk redundancies, fostering development of chronotherapeutic agents that selectively enhance acute reparative functions while suppressing chronic neurotoxicity. Ultimately, these directions will promote translational progress by bridging basic mechanistic insights with clinical applications, such as repurposing immunomodulators for TBI subtypes and improving long-term outcomes through precision medicine.

## References

[1] Capizzi A Woo J Verduzco-Gutierrez M . Traumatic brain injury: an overview of epidemiology, pathophysiology, and medical management. Med Clinics North America. (2020) 104. doi: 10.1016/j.mcna.2019.11.001, PMID: 32035565

[2] Young L Rule GT Bocchieri RT Walilko TJ Burns JM Ling G . When physics meets biology: Low and high-velocity penetration, blunt impact, and blast injuries to the brain. Front Neurol. (2015) 6:89. doi: 10.3389/fneur.2015.00089, PMID: 25999910 PMC4423508

[3] Jiang JY Gao GY Feng JF Mao Q Chen LG Yang XF . Traumatic brain injury in China. Lancet Neurol. (2019) 18:286–95. doi: 10.1016/S1474-4422(18)30469-1, PMID: 30784557

[4] Maas AIR Menon DK Adelson PD Andelic N Bell MJ Belli A . Traumatic brain injury: Integrated approaches to improve prevention, clinical care, and research. Lancet Neurol. (2017) 16. doi: 10.1016/S1474-4422(17)30371-X, PMID: 29122524

[5] Delic V Beck KD Pang KCH Citron BA . Biological links between traumatic brain injury and Parkinson’s disease. Acta Neuropathologica Commun. (2020) 8. doi: 10.1186/s40478-020-00924-7, PMID: 32264976 PMC7137235

[6] Fann JR Ribe AR Pedersen HS Fenger-Grøn M Christensen J Benros ME . Long-term risk of dementia among people with traumatic brain injury in Denmark: a population-based observational cohort study. Lancet Psychiatry. (2018) 5. doi: 10.1016/S2215-0366(18)30065-8, PMID: 29653873

[7] Shin MK Vázquez-Rosa E Koh Y Dhar M Chaubey K Cintrón-Pérez CJ . Reducing acetylated tau is neuroprotective in brain injury. Cell. (2021) 184. doi: 10.1016/j.cell.2021.03.032, PMID: 33852912 PMC8491234

[8] Jenkins PO De Simoni S Bourke NJ Fleminger J Scott G Towey DJ . Stratifying drug treatment of cognitive impairments after traumatic brain injury using neuroimaging. Brain. (2019) 142. doi: 10.1093/brain/awz149, PMID: 31199462

[9] Ko JH Yoon SO Lee HJ Oh JY . Rapamycin regulates macrophage activation by inhibiting NLRP3 inflammasome-p38 MAPK-NFκB pathways in autophagy- and p62-dependent manners. Oncotarget. (2017) 8. doi: 10.18632/oncotarget.17256, PMID: 28489580 PMC5522223

[10] Lefevre-Dognin C Cogné M Perdrieau V Granger A Heslot C Azouvi P . Definition and epidemiology of mild traumatic brain injury. Neurochirurgie. (2021) 67. doi: 10.1016/j.neuchi.2020.02.002, PMID: 32387427

[11] Popovitz J Mysore SP Adwanikar H . Long-term effects of traumatic brain injury on anxiety-like behaviors in mice: behavioral and neural correlates. Front Behav Neurosci. (2019) 13. doi: 10.3389/fnbeh.2019.00006, PMID: 30728770 PMC6351473

[12] Kumari N Bagri K Kumari S Deshmukh R . Traumatic Brian Injury (TBI) unraveled: molecular disruptions and therapeutic avenues. Inflammopharmacology. (2025) 33:4323–34. doi: 10.1007/s10787-025-01870-3, PMID: 40715927

[13] Obukohwo OM Oreoluwa OA Andrew UO Williams UE . Microglia-mediated neuroinflammation in traumatic brain injury: a review. Mol Biol Rep. (2024) 51. doi: 10.1007/s11033-024-09995-4, PMID: 39425760

[14] Gadani SP Cronk JC Norris GT Kipnis J . IL-4 in the brain: A cytokine to remember. J Immunol. (2012) 189. doi: 10.4049/jimmunol.1202246, PMID: 23087426 PMC3481177

[15] Radpour M Khoshkroodian B Asgari T Pourbadie HG Sayyah M . Interleukin 4 reduces brain hyperexcitability after traumatic injury by downregulating TNF-α, upregulating IL-10/TGF-β, and potential directing macrophage/microglia to the M2 anti-inflammatory phenotype. Inflammation. (2023) 46. doi: 10.1007/s10753-023-01843-0, PMID: 37259014

[16] Helmy A Carpenter KLH Menon DK Pickard JD Hutchinson PJA . The cytokine response to human traumatic brain injury: Temporal profiles and evidence for cerebral parenchymal production. J Cereb Blood Flow Metab. (2011) 31. doi: 10.1038/jcbfm.2010.142, PMID: 20717122 PMC3049520

[17] Slough E Pitt-Francis A Belli A Ahmed Z Di Pietro V Stevens AR . Investigating the role of neutrophil extracellular traps as a therapeutic target in traumatic brain injury: a systematic review and meta-analysis. Mol Neurobiol. (2025) 62:14923–46. doi: 10.1007/s12035-025-05053-7, PMID: 40408025 PMC12511129

[18] Feng Z Min L Liang L Chen B Chen H Zhou Y . Neutrophil extracellular traps exacerbate secondary injury via promoting neuroinflammation and blood–spinal cord barrier disruption in spinal cord injury. Front Immunol. (2021) 12. doi: 10.3389/fimmu.2021.698249, PMID: 34456910 PMC8385494

[19] Piancone F La Rosa F Hernis A Marventano I Arcuri P Rabuffetti M . Neuroinflammatory signature of post-traumatic confusional state: the role of cytokines in moderate-to-severe traumatic brain injury. Int J Mol Sci. (2025) 26. doi: 10.3390/ijms26178593, PMID: 40943512 PMC12429489

[20] Zhang H Zhang X Chai Y Wang Y Zhang J Chen X . Astrocyte-mediated inflammatory responses in traumatic brain injury: mechanisms and potential interventions. Front Immunol. (2025) 16:1584577. doi: 10.3389/fimmu.2025.1584577, PMID: 40406119 PMC12094960

[21] Vaibhav K Braun M Alverson K Khodadadi H Kutiyanawalla A Ward A . Neutrophil extracellular traps exacerbate neurological deficits after traumatic brain injury. Science Advances. (2020) 6:eaax8847. doi: 10.1126/sciadv.aax8847, PMID: 32523980 PMC7259928

[22] Simon DW McGeachy MJ Baylr H Clark RSB Loane DJ Kochanek PM . The far-reaching scope of neuroinflammation after traumatic brain injury. Nat Rev Neurol. (2017) 13. doi: 10.1038/nrneurol.2017.1, PMID: 28186177 PMC5675525

[23] Witcher KG Bray CE Chunchai T Zhao F O’Neil SM Gordillo AJ . Traumatic brain injury causes chronic cortical inflammation and neuronal dysfunction mediated by Microglia. J Neurosci. (2021) 41. doi: 10.1523/JNEUROSCI.2469-20.2020, PMID: 33452227 PMC7896020

[24] Schimmel S Acosta S Lozano D . Neuroinflammation in traumatic brain injury: A chronic response to an acute injury. Brain Circ. (2017) 3. doi: 10.4103/bc.bc_18_17, PMID: 30276315 PMC6057689

[25] Kim SY Yeh PH Ollinger JM Morris HD Hood MN Ho VB . Military-related mild traumatic brain injury: clinical characteristics, advanced neuroimaging, and molecular mechanisms. Trans Psychiatry. (2023) 13. doi: 10.1038/s41398-023-02569-1, PMID: 37652994 PMC10471788

[26] Kong LZ Zhang RL Hu SH Lai JB . Military traumatic brain injury: a challenge straddling neurology and psychiatry. Military Med Res. (2022) 9. doi: 10.1186/s40779-021-00363-y, PMID: 34991734 PMC8740337

[27] Ma X Aravind A Pfister BJ Chandra N Haorah J . Animal models of traumatic brain injury and assessment of injury severity. Mol Neurobiol. (2019) 56. doi: 10.1007/s12035-018-1454-5, PMID: 30603958

[28] Acosta SA Tajiri N Shinozuka K Ishikawa H Grimmig B Diamond D . Long-term upregulation of inflammation and suppression of cell proliferation in the brain of adult rats exposed to traumatic brain injury using the controlled cortical impact model. PloS One. (2013) 8. doi: 10.1371/annotation/a04a7468-d105-42f3-ba47-263ea2864681, PMID: 23301065 PMC3536766

[29] Iboaya A Harris JL Arickx AN Nudo RJ . Models of traumatic brain injury in aged animals: A clinical perspective. Neurorehabilitation Neural Repair. (2019) 33. doi: 10.1177/1545968319883879, PMID: 31722616 PMC6920554

[30] Bodnar CN Roberts KN Higgins EK Bachstetter AD . A systematic review of closed head injury models of mild traumatic brain injury in mice and rats. J Neurotrauma. (2019) 36. doi: 10.1089/neu.2018.612, PMID: 30661454 PMC6555186

[31] Plantman S Ng KC Lu J Davidsson J Risling M . Characterization of a novel rat model of penetrating traumatic brain injury. J Neurotrauma. (2012) 29. doi: 10.1089/neu.2011.2182, PMID: 22181060

[32] Freeman-Jones E Miller WH Work LM Fullerton JL . Polypathologies and animal models of traumatic brain injury. Brain Sci. (2023) 13. doi: 10.3390/brainsci13121709, PMID: 38137157 PMC10741988

[33] Murray KN Parry-Jones AR Allan SM . Interleukin-1 and acute brain injury. Front Cell Neurosci. (2015) 9. doi: 10.3389/fncel.2015.00018, PMID: 25705177 PMC4319479

[34] Voet S Srinivasan S Lamkanfi M van Loo G . Inflammasomes in neuroinflammatory and neurodegenerative diseases. EMBO Mol Med. (2019) 11. doi: 10.15252/emmm.201810248, PMID: 31015277 PMC6554670

[35] Vedantam A Brennan J Levin HS McCarthy JJ Dash PK Redell JB . Early versus Late Profiles of Inflammatory Cytokines after Mild Traumatic Brain Injury and Their Association with Neuropsychological Outcomes. J Neurotrauma. (2021) 38. doi: 10.1089/neu.2019.6979, PMID: 32600167 PMC7757539

[36] Flygt J Ruscher K Norberg A Mir A Gram H Clausen F . Neutralization of interleukin-1β following diffuse traumatic brain injury in the mouse attenuates the loss of mature oligodendrocytes. J Neurotrauma. (2018) 35. doi: 10.1089/neu.2018.5660, PMID: 29690837 PMC6247990

[37] Ozen I Ruscher K Nilsson R Flygt J Clausen F Marklund N . Interleukin-1 beta neutralization attenuates traumatic brain injury-induced microglia activation and neuronal changes in the globus pallidus. Int J Mol Sci. (2020) 21. doi: 10.3390/ijms21020387, PMID: 31936248 PMC7014296

[38] Newell EA Todd BP Mahoney J Pieper AA Ferguson PJ Bassuk AG . Combined blockade of interleukin-1α and -1β signaling protects mice from cognitive dysfunction after traumatic brain injury. eNeuro. (2018) 5. doi: 10.1523/ENEURO.0385-17.2018, PMID: 29662944 PMC5898697

[39] Bodnar CN Watson JB Higgins EK Quan N Bachstetter AD . Inflammatory regulation of CNS barriers after traumatic brain injury: A tale directed by interleukin-1. Front Immunol. (2021) 12:688254. doi: 10.3389/fimmu.2021.688254, PMID: 34093593 PMC8176952

[40] Cáceres E Olivella JC Di Napoli M Raihane AS Divani AA . Immune response in traumatic brain injury. Curr Neurol Neurosci Rep. (2024) 24:593–609. doi: 10.1007/s11910-024-01382-7, PMID: 39467990 PMC11538248

[41] García-Bueno B Caso JR Leza JC . Stress as a neuroinflammatory condition in brain: Damaging and protective mechanisms. Neurosci Biobehav Rev. (2008) 32. doi: 10.1016/j.neubiorev.2008.04.001, PMID: 18468686

[42] Harris F Berdugo YA Tree T . IL-2-based approaches to Treg enhancement. Clin Exp Immunol. (2023) 211. doi: 10.1093/cei/uxac105, PMID: 36399073 PMC10019135

[43] Czerpaniak K do Nascimento LF Guo T Zhang J Liu X Sarzaeim M . Low-dose interleukin-2 reverses traumatic brain injury-induced cognitive deficit and pain in a murine model. Ann Neurol. (2024) 96:508–25. doi: 10.1002/ana.26998, PMID: 39032123 PMC11324417

[44] Yshii L Pasciuto E Bielefeld P Mascali L Lemaitre P Marino M . Astrocyte-targeted gene delivery of interleukin 2 specifically increases brain-resident regulatory T cell numbers and protects against pathological neuroinflammation. Nat Immunol. (2022) 23. doi: 10.1038/s41590-022-01208-z, PMID: 35618831 PMC9174055

[45] Johnson NH Hadad R Taylor RR Pilar JR Salazar O Llompart-Pou JA . Inflammatory biomarkers of traumatic brain injury. Pharmaceuticals. (2022) 15. doi: 10.3390/ph15060660, PMID: 35745576 PMC9227014

[46] Mitchell RE Hassan M Burton BR Britton G Hill EV Verhagen J . IL-4 enhances IL-10 production in Th1 cells: Implications for Th1 and Th2 regulation. Sci Rep. (2017) 7. doi: 10.1038/s41598-017-11803-y, PMID: 28900244 PMC5595963

[47] Francos-Quijorna I Amo-Aparicio J Martinez-Muriana A López-Vales R . IL-4 drives microglia and macrophages toward a phenotype conducive for tissue repair and functional recovery after spinal cord injury. Glia. (2016) 64. doi: 10.1002/glia.23041, PMID: 27470986

[48] Pu H Zheng X Jiang X Mu H Xu F Zhu W . Interleukin-4 improves white matter integrity and functional recovery after murine traumatic brain injury via oligodendroglial PPARγ. J Cereb Blood Flow Metab. (2021) 41. doi: 10.1177/0271678X20941393, PMID: 32757740 PMC7922743

[49] Yang J Ran M Li H Lin Y Ma K Yang Y . New insight into neurological degeneration: Inflammatory cytokines and blood–brain barrier. Front Mol Neurosci. (2022) 15:1013933. doi: 10.3389/fnmol.2022.1013933, PMID: 36353359 PMC9637688

[50] Nishibori M Wang D Ousaka D Wake H . High mobility group box-1 and blood–brain barrier disruption. Cells. (2020) 9. doi: 10.3390/cells9122650, PMID: 33321691 PMC7764171

[51] Sanchis P Fernández-Gayol O Vizueta J Comes G Canal C Escrig A . Microglial cell-derived interleukin-6 influences behavior and inflammatory response in the brain following traumatic brain injury. Glia. (2020) 68. doi: 10.1002/glia.23758, PMID: 31799746

[52] Willis EF MacDonald KPA Nguyen QH Garrido AL Gillespie ER Harley SBR . Repopulating microglia promote brain repair in an IL-6-dependent manner. Cell. (2020) 180. doi: 10.1016/j.cell.2020.02.013, PMID: 32142677

[53] Wu HY Mao XF Tang XQ Ali U Apryani E Liu H . Spinal interleukin-10 produces antinociception in neuropathy through microglial β-endorphin expression, separated from antineuroinflammation. Brain Behav Immun. (2018) 73. doi: 10.1016/j.bbi.2018.06.015, PMID: 29928964

[54] Shanaki-Barvasad M Almolda B González B Castellano B . Astrocyte-targeted overproduction of IL-10 reduces neurodegeneration after TBI. Exp Neurobiol. (2022) 31., PMID: 35786640 10.5607/en21035 PMC9272120

[55] Barrett JP Henry RJ Villapol S Stoica BA Kumar A Burns MP . NOX2 deficiency alters macrophage phenotype through an IL-10/STAT3 dependent mechanism: Implications for traumatic brain injury. J Neuroinflamm. (2017) 14. doi: 10.1186/s12974-017-0843-4, PMID: 28340575 PMC5366128

[56] Lin R Chen F Wen S Teng T Pan Y Huang H . Interleukin-10 attenuates impairment of the blood-brain barrier in a severe acute pancreatitis rat model. J Inflammation (United Kingdom). (2018) 15. doi: 10.1186/s12950-018-0180-0, PMID: 29497350 PMC5828420

[57] Lagerstedt L Azurmendi L Tenovuo O Katila AJ Takala RSK Blennow K . Interleukin 10 and heart fatty acid-binding protein as early outcome predictors in patients with traumatic brain injury. Front Neurol. (2020) 11. doi: 10.3389/fneur.2020.00376, PMID: 32581990 PMC7280446

[58] Di Battista AP Rhind SG Hutchison MG Hassan S Shiu MY Inaba K . Inflammatory cytokine and chemokine profiles are associated with patient outcome and the hyperadrenergic state following acute brain injury. J Neuroinflamm. (2016) 13. doi: 10.1186/s12974-016-0500-3, PMID: 26883121 PMC4754875

[59] Lee JH Wei ZZ Cao W Won S Gu X Winter M . Regulation of therapeutic hypothermia on inflammatory cytokines, microglia polarization, migration and functional recovery after ischemic stroke in mice. Neurobiol Dis. (2016) 96. doi: 10.1016/j.nbd.2016.09.013, PMID: 27659107 PMC5161414

[60] Roquilly A David G Cinotti R Vourc’h M Morin H Rozec B . Role of IL-12 in overcoming the low responsiveness of NK cells to missing self after traumatic brain injury. Clin Immunol. (2017) 177. doi: 10.1016/j.clim.2015.08.006, PMID: 26387630

[61] Zhao Q Xie F Guo Dzhi Ju Fdi He J Yao Tting . Hydrogen inhalation inhibits microglia activation and neuroinflammation in a rat model of traumatic brain injury. Brain Res. (2020) 1748. doi: 10.1016/j.brainres.2020.147053, PMID: 32814064

[62] Rosas Almanza J Stehlik KE Page JJ Xiong SH Tabor EG Aperi B . IL-12p40 promotes secondary damage and functional impairment after spinal cord contusional injury. J Neurosci Res. (2022) 100. doi: 10.1002/jnr.25122, PMID: 36089917

[63] Kuwabara T Ishikawa F Kondo M Kakiuchi T . The role of IL-17 and related cytokines in inflammatory autoimmune diseases. Mediators Inflamm. (2017) 2017. doi: 10.1155/2017/3908061, PMID: 28316374 PMC5337858

[64] Abou-El-Hassan H Rezende RM Izzy S Gabriely G Yahya T Tatematsu BK . Vγ1 and Vγ4 gamma-delta T cells play opposing roles in the immunopathology of traumatic brain injury in males. Nat Commun. (2023) 14. doi: 10.1038/s41467-023-39857-9, PMID: 37463881 PMC10354011

[65] Amiresmaili S Khaksari M Shahrokhi N Abolhassani M . Evolution of TLR4 role in mediating the hepatoprotective effects of estradiol after traumatic brain injury in male rats. Biochem Pharmacol. (2020) 178. doi: 10.1016/j.bcp.2020.114044, PMID: 32445868

[66] Gao S-J Liu L Li D-Y Liu D-Q Zhang L-Q Wu J-Y . Interleukin-17: A putative novel pharmacological target for pathological pain. Curr Neuropharmacol. (2023) 22. doi: 10.2174/1570159x21666230811142713, PMID: 37581321 PMC10788884

[67] Cui C Zhang D Sun K Li H Xu L Lin G . Propofol maintains Th17/Treg cell balance and reduces inflammation in rats with traumatic brain injury via the miR-145-3p/NFATc2/NF-κB axis. Int J Mol Med. (2021) 48. doi: 10.3892/ijmm.2021.4968, PMID: 34036377 PMC8148094

[68] Cui W Hull L Zizzo A Wang L Lin B Zhai M . Pharmacokinetic study of rhIL-18BP and its effect on radiation-induced cytokine changes in mouse serum and intestine. Toxics. (2023) 11. doi: 10.3892/ijmm.2021.4968, PMID: 36668761 PMC9863660

[69] Cumiskey D Pickering M O’Connor JJ . Interleukin-18 mediated inhibition of LTP in the rat dentate gyrus is attenuated in the presence of mGluR antagonists. Neurosci Lett. (2007) 412. doi: 10.1016/j.neulet.2006.11.007, PMID: 17123727

[70] Dinarello CA Novick D Kim S Kaplanski G . Interleukin-18 and IL-18 binding protein. Front Immunol. (2013) 4:289. doi: 10.3389/fimmu.2013.00289, PMID: 24115947 PMC3792554

[71] Lu F Lan Z Xin Z He C Guo Z Xia X . Emerging insights into molecular mechanisms underlying pyroptosis and functions of inflammasomes in diseases. J Cell Physiol. (2020) 235. doi: 10.1002/jcp.29268, PMID: 31621910 PMC13482838

[72] Qiu Z Lei S Zhao B Wu Y Su W Liu M . NLRP3 inflammasome activation-mediated pyroptosis aggravates myocardial ischemia/reperfusion injury in diabetic rats. Oxid Med Cell Longev. (2017) 2017. doi: 10.1155/2017/9743280, PMID: 29062465 PMC5618779

[73] Bortolotti P Faure E Kipnis E . Inflammasomes in tissue damages and immune disorders after trauma. Front Immunol. (2018) 9:1900. doi: 10.3389/fimmu.2018.01900, PMID: 30166988 PMC6105702

[74] Ciaramella A Della Vedova C Salani F Viganotti M D’Ippolito M Caltagirone C . Increased levels of serum IL-18 are associated with the long-term outcome of severe traumatic brain injury. Neuroimmunomodulation. (2013) 21. doi: 10.1159/000354764, PMID: 24080899

[75] Kopitar-Jerala N . The role of interferons in inflammation and inflammasome activation. Front Immunol. (2017) 8:873. doi: 10.3389/fimmu.2017.00873, PMID: 28791024 PMC5525294

[76] Clarkson BDS Kahoud RJ McCarthy CB Howe CL . Inflammatory cytokine-induced changes in neural network activity measured by waveform analysis of high-content calcium imaging in murine cortical neurons. Sci Rep. (2017) 7. doi: 10.1038/s41598-017-09182-5, PMID: 28831096 PMC5567248

[77] Roselli F Chandrasekar A Morganti-Kossmann MC . Interferons in traumatic brain and spinal cord injury: Current evidence for translational application. Front Neurol. (2018) 9:458. doi: 10.3389/fneur.2018.00458, PMID: 29971040 PMC6018073

[78] Packer JM Bray CE Beckman NB Wangler LM Davis AC Goodman EJ . Impaired cortical neuronal homeostasis and cognition after diffuse traumatic brain injury are dependent on microglia and type I interferon responses. Glia. (2024) 72. doi: 10.1002/glia.24475, PMID: 37937831 PMC10764078

[79] Abd-El-Basset EM Rao MS Alsaqobi A . Interferon-gamma and interleukin-1Beta enhance the secretion of brain-derived neurotrophic factor and promotes the survival of cortical neurons in brain injury. Neurosci Insights. (2020) 15. doi: 10.1177/2633105520947081, PMID: 32776009 PMC7391446

[80] Chaban V Clarke GJB Skandsen T Islam R Einarsen CE Vik A . Systemic inflammation persists the first year after mild traumatic brain injury: results from the prospective trondheim mild traumatic brain injury study. J Neurotrauma. (2020) 37. doi: 10.1089/neu.2019.6963, PMID: 32326805 PMC7502683

[81] Qin H Qin J Hu J Huang H Ma L . Malva sylvestris attenuates cognitive deficits in a repetitive mild traumatic brain injury rat model by reducing neuronal degeneration and astrocytosis in the hippocampus. Med Sci Monitor. (2017) 23. doi: 10.12659/MSM.905429, PMID: 29276216 PMC5749139

[82] Santarsieri M Kumar RG Kochanek PM Berga S Wagner AK . Variable neuroendocrine-immune dysfunction in individuals with unfavorable outcome after severe traumatic brain injury. Brain Behav Immun. (2015) 45. doi: 10.1016/j.bbi.2014.09.003, PMID: 25218898 PMC4342288

[83] Choudhary A Varshney R Kumar A . & Kaushik, K. A prospective study of novel therapeutic targets interleukin 6, tumor necrosis factor α, and interferon γ as predictive biomarkers for the development of posttraumatic epilepsy. World Neurosurg X. (2021) 12. doi: 10.1016/j.wnsx.2021.100107, PMID: 34195601 PMC8233159

[84] Ding X Sun X Shen Xfang Lu Y Wang Jqiang Sun Zrong . Propofol attenuates TNF-α-induced MMP-9 expression in human cerebral microvascular endothelial cells by inhibiting Ca2+/CAMK II/ERK/NF-κB signaling pathway. Acta Pharmacol Sin. (2019) 40. doi: 10.1038/s41401-019-0258-0, PMID: 31235816 PMC6786358

[85] Zheng S Wang C Lin L Mu S Liu H Hu X . TNF-a Impairs Pericyte-Mediated Cerebral Microcirculation via the NF-jB/iNOS Axis after Experimental Traumatic Brain Injury. J Neurotrauma. (2023) 40. doi: 10.1038/s41401-019-0258-0, PMID: 35972751

[86] Shao X Yang X Shen J Chen S Jiang X Wang Q . TNF-α–induced p53 activation induces apoptosis in neurological injury. J Cell Mol Med. (2020) 24. doi: 10.1111/jcmm.15333, PMID: 32344470 PMC7299703

[87] Kim M Jung K Ko Y Kim IS Hwang K Jang JH . TNF-α pretreatment improves the survival and function of transplanted human neural progenitor cells following hypoxic-ischemic brain injury. Cells. (2020) 9. doi: 10.3390/cells9051195, PMID: 32403417 PMC7291333

[88] Luo C Yang J Liu Z Jing D . Predicting the recurrence and overall survival of patients with glioma based on histopathological images using deep learning. Front Neurol. (2023) 14. doi: 10.3389/fneur.2023.1100933, PMID: 37064206 PMC10102594

[89] Li Z Xiao J Xu X Li W Zhong R Qi L . M-CSF, IL-6, and TGF-β promote generation of a new subset of tissue repair macrophage for traumatic brain injury recovery. Sci Adv. (2021) 7. doi: 10.1126/sciadv.abb6260, PMID: 33712456 PMC7954455

[90] Li Y Chen W Deng H Li T Liu Z Liu X . TGF-β1 Protects Trauma-injured Murine Cortical Neurons by Upregulating L-type Calcium Channel Cav1.2 via the p38 Pathway. Neuroscience. (2022) 492. doi: 10.1016/j.neuroscience.2022.04.010, PMID: 35460836

[91] Zhao J Wang B Wu X Yang Z Huang T Guo X . TGFβ1 alleviates axonal injury by regulating microglia/macrophages alternative activation in traumatic brain injury. Brain Res Bull. (2020) 161. doi: 10.1016/j.brainresbull.2020.04.011, PMID: 32389801

[92] Luo J . TGF-β as a key modulator of astrocyte reactivity: disease relevance and therapeutic implications. Biomedicines. (2022) 10. doi: 10.3390/biomedicines10051206, PMID: 35625943 PMC9138510

[93] Xie Y Chen X Li Y Chen S Liu S Yu Z . Transforming growth factor-β1 protects against LPC-induced cognitive deficit by attenuating pyroptosis of microglia via NF-κB/ERK1/2 pathways. J Neuroinflamm. (2022) 19. doi: 10.1186/s12974-022-02557-0, PMID: 35902863 PMC9336072

[94] Bhowmick S Alikunju S Abdul-Muneer PM . NADPH oxidase-induced activation of transforming growth factor-beta-1 causes neuropathy by suppressing antioxidant signaling pathways in alcohol use disorder. Neuropharmacology. (2022) 213. doi: 10.1016/j.neuropharm.2022.109136, PMID: 35584723

[95] Patel RK Prasad N Kuwar R Haldar D Abdul-Muneer PM . Transforming growth factor-beta 1 signaling regulates neuroinflammation and apoptosis in mild traumatic brain injury. Brain Behav Immun. (2017) 64:244–58. doi: 10.1016/j.bbi.2017.04.012, PMID: 28433746

[96] Liu XL Sun DD Zheng MT Li XT Niu HH Zhang L . Maraviroc promotes recovery from traumatic brain injury in mice by suppression of neuroinflammation and activation of neurotoxic reactive astrocytes. Neural Regen Res. (2023) 18. doi: 10.4103/1673-5374.344829, PMID: 35799534 PMC9241405

[97] Lv R Du L Liu X Zhou F Zhang Z Zhang L . Rosmarinic acid attenuates inflammatory responses through inhibiting HMGB1/TLR4/NF-κB signaling pathway in a mouse model of Parkinson’s disease. Life Sci. (2019) 223. doi: 10.1016/j.lfs.2019.03.030, PMID: 30880023

[98] Fan H Tang HB Chen Z Wang HQ Zhang L Jiang Y . Inhibiting HMGB1-RAGE axis prevents pro-inflammatory macrophages/microglia polarization and affords neuroprotection after spinal cord injury. J Neuroinflamm. (2020) 17. doi: 10.1186/s12974-020-01973-4, PMID: 33036632 PMC7547440

[99] Nishibori M Mori S Takahashi HK . Anti-HMGB1 monoclonal antibody therapy for a wide range of CNS and PNS diseases. J Pharmacol Sci. (2019) 140. doi: 10.1016/j.jphs.2019.04.006, PMID: 31105025

[100] Okuma Y Liu K Wake H Liu R Nishimura Y Hui Z . Glycyrrhizin inhibits traumatic brain injury by reducing HMGB1-RAGE interaction. Neuropharmacology. (2014) 85. doi: 10.1016/j.neuropharm.2014.05.007, PMID: 24859607

[101] Au AK Aneja RK Bell MJ Bayir H Feldman K Adelson PD . Cerebrospinal fluid levels of high-mobility group box 1 and cytochrome C predict outcome after pediatric traumatic brain injury. J Neurotrauma. (2012) 29. doi: 10.1089/neu.2011.2171, PMID: 22540160 PMC3408241

[102] Tan SW Zhao Y Li P Ning YL Huang ZZ Yang N . HMGB1 mediates cognitive impairment caused by the NLRP3 inflammasome in the late stage of traumatic brain injury. J Neuroinflamm. (2021) 18. doi: 10.1186/s12974-021-02274-0, PMID: 34666797 PMC8527642

[103] Ved R Sharouf F Harari B Muzaffar M Manivannan S Ormonde C . Disulfide HMGB1 acts via TLR2/4 receptors to reduce the numbers of oligodendrocyte progenitor cells after traumatic injury in *vitro*. Sci Rep. (2021) 11. doi: 10.1038/s41598-021-84932-0, PMID: 33731757 PMC7971069

[104] Manivannan S Wales E Zaben M . The role of HMGB1 in traumatic brain injury—Bridging the gap between the laboratory and clinical studies. Curr Neurol Neurosci Rep. (2021) 21. doi: 10.1007/s11910-021-01158-3, PMID: 34870759

[105] Vinh To X Mohamed AZ Cumming P Nasrallah FA . Subacute cytokine changes after a traumatic brain injury predict chronic brain microstructural alterations on advanced diffusion imaging in the male rat. Brain Behav Immun. (2022) 102. doi: 10.1016/j.bbi.2022.02.017, PMID: 35183698

[106] Williams AM Wu Z Bhatti UF Biesterveld BE Kemp MT Wakam GK . Early single-dose exosome treatment improves neurologic outcomes in a 7-day swine model of traumatic brain injury and hemorrhagic shock. J Trauma Acute Care Surg. (2020) 89. doi: 10.1097/TA.0000000000002698, PMID: 32218019

[107] Sribnick EA Warner T Hall MW . Granulocyte- macrophage colony-stimulating factor reverses immunosuppression acutely following a traumatic brain injury and hemorrhage polytrauma in a juvenile male rat model. J Neurotrauma. (2024) 41:e1710–20. doi: 10.1089/neu.2023.0169, PMID: 38623766 PMC11564832

[108] Vink R Corrigan F . Chronic traumatic encephalopathy: Genes load the gun and repeated concussion pulls the trigger. Neural Regen Res. (2022) 17. doi: 10.4103/1673-5374.335147, PMID: 35142676 PMC8848591

[109] Chen Y Wang Y Xu J Hou T Zhu J Jiang Y . Multiplex assessment of serum chemokines CCL2, CCL5, CXCL1, CXCL10, and CXCL13 following traumatic brain injury. Inflammation. (2023) 46. doi: 10.1007/s10753-022-01729-7, PMID: 35969281

[110] Gyoneva S Ransohoff RM . Inflammatory reaction after traumatic brain injury: Therapeutic potential of targeting cell-cell communication by chemokines. Trends Pharmacol Sci. (2015) 36. doi: 10.1016/j.tips.2015.04.003, PMID: 25979813 PMC4485943

[111] Sharma R Chu E Dill LK Shad A Zamani A O’Brien TJ . Ccr2 gene ablation does not influence seizure susceptibility, tissue damage, or cellular inflammation after murine pediatric traumatic brain injury. J Neurotrauma. (2023) 40. doi: 10.1089/neu.2022.0033, PMID: 36070444

[112] Thangavel R Kaur H Dubova I Selvakumar GP Ahmed ME Raikwar SP . Parkinson’s disease dementia patients: expression of glia maturation factor in the brain. Int J Mol Sci. (2024) 25. doi: 10.3390/ijms25021182, PMID: 38256254 PMC11154259

[113] Ahmed ME Selvakumar GP Kempuraj D Raikwar SP Thangavel R Bazley K . Glia maturation factor (GMF) regulates microglial expression phenotypes and the associated neurological deficits in a mouse model of traumatic brain injury. Mol Neurobiol. (2020) 57. doi: 10.1007/s12035-020-02040-y, PMID: 32737763

[114] Raikwar SP Thangavel R Ahmed ME Selvakumar GP Kempuraj D Wu K . Real-time noninvasive bioluminescence, ultrasound and photoacoustic imaging in NFκB-RE-luc transgenic mice reveal glia maturation factor-mediated immediate and sustained spatio-temporal activation of NFκB signaling post-traumatic brain injury in a gender-specific manner. Cell Mol Neurobiol. (2021) 41. doi: 10.1007/s10571-020-00937-9, PMID: 32785863 PMC8188847

[115] Yin G Du M Li R Li K Huang X Duan D . Glia maturation factor beta is required for reactive gliosis after traumatic brain injury in zebrafish. Exp Neurol. (2018) 305. doi: 10.1016/j.expneurol.2018.04.008, PMID: 29655639

[116] Sciacchitano S Lavra L Morgante A Ulivieri A Magi F De Francesco GP . Galectin-3: One molecule for an alphabet of diseases, from A to Z. Int J Mol Sci. (2018) 19. doi: 10.3390/ijms1902037, PMID: 29373564 PMC5855601

[117] Mostacada K Oliveira FL Villa-Verde DMS Martinez AMB . Lack of galectin-3 improves the functional outcome and tissue sparing by modulating inflammatory response after a compressive spinal cord injury. Exp Neurol. (2015) 271. doi: 10.1016/j.expneurol.2015.07.006, PMID: 26183316

[118] Pajoohesh-Ganji A Knoblach SM Faden AI Byrnes KR . Characterization of inflammatory gene expression and galectin-3 function after spinal cord injury in mice. Brain Res. (2012) 1475. doi: 10.1016/j.brainres.2012.07.058, PMID: 22884909 PMC3433585

[119] Shan R Szmydynger-Chodobska J Warren OU Mohammad F Zink BJ Chodobski A . A new panel of blood biomarkers for the diagnosis of mild traumatic brain injury/concussion in adults. J Neurotrauma. (2016) 33. doi: 10.1089/neu.2014.3811, PMID: 25794137

[120] Yip PK Carrillo-Jimenez A King P Vilalta A Nomura K Chau CC . Galectin-3 released in response to traumatic brain injury acts as an alarmin orchestrating brain immune response and promoting neurodegeneration. Sci Rep. (2017) 7. doi: 10.1038/srep41689, PMID: 28128358 PMC5269662

[121] Venkatesan C Chrzaszcz MA Choi N Wainwright MS . Chronic upregulation of activated microglia immunoreactive for galectin-3/Mac-2 and nerve growth factor following diffuse axonal injury. J Neuroinflamm. (2010) 7. doi: 10.1186/1742-2094-7-32, PMID: 20507613 PMC2891720

[122] Ribeiro TN Delgado-García LM Porcionatto MA . Notch1 and galectin-3 modulate cortical reactive astrocyte response after brain injury. Front Cell Dev Biol. (2021) 9. doi: 10.3389/fcell.2021.649854, PMID: 34222228 PMC8244823

[123] Shen YF Yu WH Dong XQ Du Q Yang DB Wu GQ . The change of plasma galectin-3 concentrations after traumatic brain injury. Clinica Chimica Acta. (2016) 456. doi: 10.1016/j.cca.2016.02.029, PMID: 26944570

[124] Miñambres E Cemborain A Sánchez-Velasco P Gandarillas M Díaz-Regañón G Sánchez-González U . Correlation between transcranial interleukin-6 gradient and outcome in patients with acute brain injury. Crit Care Med. (2003) 31. doi: 10.1097/01.CCM.0000055370.66389.59, PMID: 12627008

[125] Nitta ME Savitz J Nelson LD Teague TK Hoelzle JB McCrea MA . Acute elevation of serum inflammatory markers predicts symptom recovery after concussion. Neurology. (2019) 93. doi: 10.1212/WNL.0000000000007864, PMID: 31270219 PMC6693429

[126] Singhal A Baker AJ Hare GMT Reinders FX Schlichter LC Moulton RJ . Association between cerebrospinal fluid interleukin-6 concentrations and outcome after severe human traumatic brain injury. J Neurotrauma. (2002) 19. doi: 10.1089/089771502320317087, PMID: 12225653

[127] Hergenroeder GW Moore AN McCoy JP Samsel L Ward NH Clifton GL . Serum IL-6: A candidate biomarker for intracranial pressure elevation following isolated traumatic brain injury. J Neuroinflamm. (2010) 7. doi: 10.1186/1742-2094-7-19, PMID: 20222971 PMC2853529

[128] Gebhard F Pfetsch H Steinbach G Strecker W Kinzl L Brückner UB . Is interleukin 6 an early marker of injury severity following major trauma in humans? Arch Surg. (2000) 135. doi: 10.1001/archsurg.135.3.291, PMID: 10722030

[129] Hang CH Shi JX Li JS Li WQ Wu W . Expressions of intestinal NF-κB, TNF-α, and IL-6 following traumatic brain injury in rats. J Surg Res. (2005) 123. doi: 10.1016/j.jss.2004.08.002, PMID: 15680377

[130] Taupin V Toulmond S Serrano A Benavides J Zavala F . Increase in IL-6, IL-1 and TNF levels in rat brain following traumatic lesion. Influence of pre- and post-traumatic treatment with Ro5 4864, a peripheral-type (p site) benzodiazepine ligand. J Neuroimmunol. (1993) 42. doi: 10.1016/0165-5728(93)90008-M, PMID: 8429103

[131] Schneider Soares FM Menezes De Souza N Librio Schwarzbold M Paim Diaz A Costa Nunes J Hohl A . Interleukin-10 is an independent biomarker of severe traumatic brain injury prognosis. Neuroimmunomodulation. (2012) 19. doi: 10.1159/000342141, PMID: 23075771

[132] Tsitsipanis C Miliaraki M Paflioti E Lazarioti S Moustakis N Ntotsikas K . Inflammation biomarkers IL-6 and IL-10 may improve the diagnostic and prognostic accuracy of currently authorized traumatic brain injury tools. Exp Ther Med. (2023) 26. doi: 10.3892/etm.2023.12063, PMID: 37408863 PMC10318605

[133] Yue JK Kobeissy FH Jain S Sun X Phelps RRL Korley FK . Neuroinflammatory biomarkers for traumatic brain injury diagnosis and prognosis: A TRACK-TBI pilot study. Neurotrauma Rep. (2023) 4:171–83. doi: 10.1089/neur.2022.0060, PMID: 36974122 PMC10039275

[134] Zhang D Zhuang D Li T Liu X Zhang Z Zhu L . An analysis of neutrophil-to-lymphocyte ratios and monocyte-to-lymphocyte ratios with six-month prognosis after cerebral contusions. Front Immunol. (2024) 15. doi: 10.3389/fimmu.2024.1336862, PMID: 38545111 PMC10967015

[135] Zhuang D Sheng J Peng G Li T Cai S Din F . Neutrophil to lymphocyte ratio predicts early growth of traumatic intracerebral haemorrhage. Ann Clin Transl Neurol. (2021) 8:1601–9. doi: 10.1002/acn3.51409, PMID: 34165245 PMC8351393

[136] Sheng J Li T Zhuang D Cai S Yang J Ding F . The Monocyte-to-Lymphocyte Ratio at Hospital Admission Is a Novel Predictor for Acute Traumatic Intraparenchymal Hemorrhage Expansion after Cerebral Contusion. Mediators Inflamm. (2020) 2020. doi: 10.1155/2020/5483981, PMID: 33456370 PMC7785383

[137] Sheng J Chen W Zhuang D Li T Yang J Cai S . A clinical predictive nomogram for traumatic brain parenchyma hematoma progression. Neurol Ther. (2022) 11. doi: 10.1007/s40120-021-00306-8, PMID: 34855160 PMC8857351

[138] Samanta RJ Chiollaz AC Needham E Yue JK Helmy A Zanier ER . Parsimonious immune-response endotypes and global outcome in patients with traumatic brain injury. EBioMedicine. (2024) 108. doi: 10.1016/j.ebiom.2024.105310, PMID: 39293212 PMC11424973

[139] Lagares A de la Cruz J Terrisse H Mejan O Pavlov V Vermorel C . An automated blood test for glial fibrillary acidic protein (GFAP) and ubiquitin carboxy-terminal hydrolase L1 (UCH-L1) to predict the absence of intracranial lesions on head CT in adult patients with mild traumatic brain injury: BRAINI, a multicentre observational study in Europe. EBioMedicine. (2024) 110. doi: 10.1016/j.ebiom.2024.105477, PMID: 39612652 PMC11647500

[140] Werner JK Albrecht J Capaldi VF Jain S Sun X Mukherjee P . Association of biomarkers of neuronal injury and inflammation with insomnia trajectories after traumatic brain injury A TRACK-TBI study. Neurology. (2024) 102. doi: 10.1212/WNL.0000000000209269, PMID: 38547447 PMC11210587

[141] Chen X Ning Y Wang B Qin J Li C Gao R . HET0016 inhibits neuronal pyroptosis in the immature brain post-TBI via the p38 MAPK signaling pathway. Neuropharmacology. (2023) 239. doi: 10.1016/j.neuropharm.2023.109687, PMID: 37579871

[142] Li G Duan L Yang F Yang L Deng Y Yu Y . Curcumin suppress inflammatory response in traumatic brain injury via p38/MAPK signaling pathway. Phytother Res. (2022) 36. doi: 10.1002/ptr.7391, PMID: 35080289

[143] Liu R Sun L Shi X Li C Guo X Wang Y . Increased expression of KNa1.2 channel by MAPK pathway regulates neuronal activity following traumatic brain injury. Neurochem Res. (2024) 49. doi: 10.1007/s11064-023-04044-1, PMID: 37875713

[144] Huang L Fang H Cheng H Mei S Cheng Y Lv Y . Epigenetic modulation of the MAPK pathway prevents isoflurane-induced neuronal apoptosis and cognitive decline in aged rats. Exp Ther Med. (2020) 20. doi: 10.3892/etm.2020.9162, PMID: 32952626 PMC7480129

[145] Otani N Nawashiro H Fukui S Nomura N Yano A Miyazawa T . Differential activation of mitogen-activated protein kinase pathways after traumatic brain injury in the rat hippocampus. J Cereb Blood Flow Metab. (2002) 22. doi: 10.1097/00004647-200203000-00010, PMID: 11891438

[146] Kodali M Madhu LN Reger RL Milutinovic B Upadhya R Gonzalez JJ . Intranasally administered human MSC-derived extracellular vesicles inhibit NLRP3-p38/MAPK signaling after TBI and prevent chronic brain dysfunction. Brain Behav Immun. (2023) 108. doi: 10.1016/j.bbi.2022.11.014, PMID: 36427808 PMC9974012

[147] Zusso M Lunardi V Franceschini D Pagetta A Lo R Stifani S . Ciprofloxacin and levofloxacin attenuate microglia inflammatory response via TLR4/NF-kB pathway. J Neuroinflamm. (2019) 16. doi: 10.1186/s12974-019-1538-9, PMID: 31319868 PMC6637517

[148] Li YF Ren X Zhang L Wang YH Chen T . Microglial polarization in TBI: Signaling pathways and influencing pharmaceuticals. Front Aging Neurosci. (2022) 14:901117. doi: 10.3389/fnagi.2022.901117, PMID: 35978950 PMC9376354

[149] El Baassiri MG Rahal SS Fulton WB Sodhi CP Hackam DJ Nasr IW . Pharmacologic Toll-like receptor 4 inhibition skews toward a favorable A1/A2 astrocytic ratio improving neurocognitive outcomes following traumatic brain injury. J Trauma Acute Care Surg. (2023) 95. doi: 10.1097/TA.0000000000003887, PMID: 36728129

[150] Yang X Zhang J Duan L Xiong H Jiang Y Liang H . Microglia activation mediated by toll-like receptor-4 impairs brain white matter tracts in rats. J Biomed Res. (2018) 32. doi: 10.7555/JBR.32.20170033, PMID: 29358565 PMC5895568

[151] Zhu H Bian C Yuan JC Chu WH Xiang X Chen F . Curcumin attenuates acute inflammatory injury by inhibiting the TLR4/MyD88/NF-κB signaling pathway in experimental traumatic brain injury. J Neuroinflamm. (2014) 11. doi: 10.1186/1742-2094-11-59, PMID: 24669820 PMC3986937

[152] Chen X Chen C Fan S Wu S Yang F Fang Z . Omega-3 polyunsaturated fatty acid attenuates the inflammatory response by modulating microglia polarization through SIRT1-mediated deacetylation of the HMGB1/NF-κB pathway following experimental traumatic brain injury. J Neuroinflamm. (2018) 15. doi: 10.1186/s12974-018-1151-3, PMID: 29678169 PMC5909267

[153] Hou Q Chen H Liu Q Yan X . FGF10 attenuates experimental traumatic brain injury through TLR4/myD88/NF-κB pathway. Cells Tissues Organs. (2021) 209:248–56. doi: 10.1159/000511381, PMID: 33440393

[154] Jiang H Yang X Wang Y Zhou C . Vitamin D Protects against Traumatic Brain Injury via Modulating TLR4/MyD88/NF- κ B Pathway-Mediated Microglial Polarization and Neuroinflammation. BioMed Res Int. (2022) 2022. doi: 10.1155/2022/3363036, PMID: 35872863 PMC9307360

[155] Feng Y Ju Y Wu Q Sun G Yan Z . TAK-242, a toll-like receptor 4 antagonist, against brain injury by alleviates autophagy and inflammation in rats. Open Life Sci. (2023) 18. doi: 10.1515/biol-2022-0662, PMID: 37528888 PMC10389675

[156] Wang XY Ba YC Xiong LL Li XL Zou Y Zhu YC . Endogenous TGFβ1 plays a crucial role in functional recovery after traumatic brain injury associated with smad3 signal in rats. Neurochem Res. (2015) 40. doi: 10.1007/s11064-015-1634-x, PMID: 26253398

[157] Chen X Yao J Lai J Lin L Chen Y Lin Y . ADAM17 aggravates the inflammatory response by modulating microglia polarization through the TGF-β1/smad pathway following experimental traumatic brain injury. J Neurotrauma. (2023) 40. doi: 10.1089/neu.2022.0373, PMID: 37029898

[158] Huang S Houghton PJ . Targeting mTOR signaling for cancer therapy. Curr Opin Pharmacol. (2003) 3. doi: 10.1016/S1471-4892(03)00071-7, PMID: 12901945

[159] Xie Y Shi X Sheng K Han G Li W Zhao Q . PI3K/Akt signaling transduction pathway, erythropoiesis and glycolysis in hypoxia (Review). Mol Med Rep. (2019) 19. doi: 10.3892/mmr.2018.9713, PMID: 30535469 PMC6323245

[160] Zhang H Xing Z Zheng J Shi J Cui C . Ursolic acid ameliorates traumatic brain injury in mice by regulating microRNA-141-mediated PDCD4/PI3K/AKT signaling pathway. Int Immunopharmacol. (2023) 120. doi: 10.1016/j.intimp.2023.110258, PMID: 37244112

[161] Zhang J Gu Y Sun W Yu L Li T . Tetrahydrocurcumin protects against GSK3β/PTEN/PI3K/akt-mediated neuroinflammatory responses and microglial polarization following traumatic brain injury. Mol Neurobiol. (2024) 61. doi: 10.1007/s12035-024-04034-6, PMID: 38368289

[162] Yang H Hong Y Gong M Cai S Yuan Z Feng S . Fisetin exerts neuroprotective effects *in vivo* and *in vitro* by inhibiting ferroptosis and oxidative stress after traumatic brain injury. . Front Pharmacol. (2024) 15. doi: 10.3389/fphar.2024.1480345, PMID: 39635435 PMC11615404

[163] Zhang Y He Z Hu Q Liu H Wen R Ru N . MiR-3571 modulates traumatic brain injury by regulating the PI3K/AKT signaling pathway via Fbxo31. Cell Biochem Biophys. (2024) 82:3629–43. doi: 10.1007/s12013-024-01452-0, PMID: 39080190

[164] Wang M Zhao R Su Y Zhai D Liang H Zhang L . 4,4′-dimethoxychalcone mitigates neuroinflammation following traumatic brain injury through modulation of the TREM2/PI3K/AKT/NF-κB signaling pathway. Inflammation. (2025) 48:3487–505. doi: 10.1007/s10753-025-02279-4, PMID: 40261458 PMC12596308

[165] Shim SS Stutzmann GE . Inhibition of glycogen synthase kinase-3: An emerging target in the treatment of traumatic brain injury. J Neurotrauma. (2016) 33. doi: 10.1089/neu.2015.4177, PMID: 26979735

[166] Amlerova Z Chmelova M Anderova M Vargova L . Reactive gliosis in traumatic brain injury: a comprehensive review. Front Cell Neurosci. (2024) 18:1335849. doi: 10.3389/fncel.2024.1335849, PMID: 38481632 PMC10933082

[167] Hung SY Chung HY Luo ST Chu YT Chen YH MacDonald IJ . Electroacupuncture improves TBI dysfunction by targeting HDAC overexpression and BDNF-associated Akt/GSK-3β signaling. Front Cell Neurosci. (2022) 16. doi: 10.3389/fncel.2022.880267, PMID: 36016833 PMC9396337

[168] Chen Y Li D Li N Loh PY Guo Y Hu X . Role of nerve signal transduction and neuroimmune crosstalk in mediating the analgesic effects of acupuncture for neuropathic pain. Front Neurol. (2023) 14:1093849. doi: 10.3389/fneur.2023.1093849, PMID: 36756246 PMC9899820

[169] Bahuguna D Amulya E Arya S Loharkar S Vambhurkar G Bhattacharjee S . Unlocking therapeutic potential in traumatic brain injury: exploring microenvironmental targets, signaling pathways and translational hurdles. Inflammopharmacology. (2025) 33:5113–44. doi: 10.1007/s10787-025-01923-7, PMID: 40889011

[170] Häcker H Karin M . Regulation and function of IKK and IKK-related kinases. Science’s STKE : Signal transduction knowledge Environ. (2006) 2006. doi: 10.1126/stke.3572006re13, PMID: 17047224

[171] Hu J Wang X Chen X Fang Y Chen K Peng W . Hydroxychloroquine attenuates neuroinflammation following traumatic brain injury by regulating the TLR4/NF-κB signaling pathway. J Neuroinflamm. (2022) 19. doi: 10.1186/s12974-022-02430-0, PMID: 35346242 PMC8961949

[172] Kalra S Malik R Singh G Bhatia S Al-Harrasi A Mohan S . Pathogenesis and management of traumatic brain injury (TBI): role of neuroinflammation and anti-inflammatory drugs. Inflammopharmacology. (2022) 30. doi: 10.1007/s10787-022-01017-8, PMID: 35802283 PMC9293826

[173] Javalgekar M Jupp B Vivash L O’Brien TJ Wright DK Jones NC . Inflammasomes at the crossroads of traumatic brain injury and post-traumatic epilepsy. J Neuroinflamm. (2024) 21. doi: 10.1186/s12974-024-03167-8, PMID: 39014496 PMC11250980

[174] Ismael S Ahmed H Adris T Parveen K Thakor P Ishrat T . The NLRP3 inflammasome: A potential therapeutic target for traumatic brain injury. Neural Regeneration Res. (2021) 16. doi: 10.4103/1673-5374.286951, PMID: 32788447 PMC7818859

[175] Zusman BE Wu Y Kochanek PM Vagni VE Janesko-Feldman K Gerzanich V . Precision effects of glibenclamide on MRI endophenotypes in clinically relevant murine traumatic brain injury. Crit Care Med. (2023) 51. doi: 10.1097/CCM.0000000000005749, PMID: 36661464 PMC9848216

[176] Chen B Shi G Xu J Zhang X Zhu Y Li L . IL-23 promotes neuronal ferroptosis via IL-23R/STAT3 signaling after traumatic brain injury. Cell Communication Signaling. (2025) 23. doi: 10.1186/s12964-025-02319-4, PMID: 40598174 PMC12219932

[177] Veillette C Umana M Gagnon MA Costerousse O Zarychanski R McAuley DF . Effect of statins on neurological functional outcomes in critically ill adult patients with traumatic brain injury: a systematic review and meta-analysis. BMJ Open. (2025) 15. doi: 10.1136/bmjopen-2024-091971, PMID: 39971597 PMC11840907

[178] Li M Huo X Wang Y Li W Xiao L Jiang Z . Effect of drug therapy on nerve repair of moderate-severe traumatic brain injury: A network meta-analysis. Front Pharmacol. (2022) 13:1021653. doi: 10.3389/fphar.2022.1021653, PMID: 36408253 PMC9666493

[179] Azad TD Shah PP Kim HB Stevens RD . Endotypes and the path to precision in moderate and severe traumatic brain injury. Neurocritical Care. (2022) 37. doi: 10.1007/s12028-022-01475-6, PMID: 35314969

[180] Wu J Ren R Chen T Su L Tang T . Neuroimmune and neuroinflammation response for traumatic brain injury. Brain Res Bull. (2024) 217. doi: 10.1016/j.brainresbull.2024.11106 39241894

[181] Bagnato S Boccagni C . Moderate/severe traumatic brain injury as a trigger of chronic neurodegeneration in humans. Neural Regeneration Res. (2020) 15. doi: 10.4103/1673-5374.272574, PMID: 31960805 PMC7047794

[182] Chen W Sheng J Guo J Peng G Hong J Li B . Cytokine cascades induced by mechanical trauma injury alter voltage-gated sodium channel activity in intact cortical neurons. J Neuroinflamm. (2017) 14:73. doi: 10.1186/s12974-017-0847-0, PMID: 28359334 PMC5374609

[183] Procès A Alpizar YA Halliez S Broné B Saudou F Ris L . Stretch-injury promotes microglia activation with enhanced phagocytic and synaptic stripping activities. Biomaterials. (2024) 305. doi: 10.1016/j.biomaterials.2023.122426, PMID: 38134473

[184] Davis AC Godbout JP . Neuroimmune dynamics and cytokines in traumatic brain injury. Trends Immunol. (2025) 47:77–91. doi: 10.1016/j.it.2025.09.009, PMID: 41130893

[185] Pathak D Sriram K . Neuron-astrocyte omnidirectional signaling in neurological health and disease. Front Mol Neurosci. (2023) 16:116932. doi: 10.3389/fnmol.2023.116932, PMID: 37363320 PMC10286832

[186] Chen W Sheng J Guo J Gao F Zhao X Dai J . Tumor necrosis factor-alpha enhances voltage-gated Na(+) currents in primary culture of mouse cortical neurons. J Neuroinflamm. (2015) 12:126. doi: 10.1186/s12974-015-0349-x, PMID: 26112872 PMC4510892

[187] Xia W Peng GY Sheng JT Zhu FF Guo JF Chen WQ . Neuroprotective effect of interleukin-6 regulation of voltage-gated Na+ channels of cortical neurons is time- and dose-dependent. Neural Regen Res. (2015) 10. doi: 10.4103/1673-5374.155436, PMID: 26170823 PMC4424755

[188] Başkurt O . Temporal dynamics of inflammatory, glial, and metabolic biomarkers following severe diffuse traumatic brain injury in a rat model. Biomedicines. (2025) 13. doi: 10.3390/biomedicines13123123, PMID: 41463131 PMC12730376

[189] Ren Z Li T Liu X Zhang Z Chen X Chen W . Transforming growth factor-beta 1 enhances discharge activity of cortical neurons. Neural Regen Res. (2025) 20. doi: 10.4103/NRR.NRR-D-23-00756, PMID: 38819066 PMC11317929

[190] Sica A Mantovani A . Macrophage plasticity and polarization: *In vivo* veritas. J Clin Invest. (2012) 122. doi: 10.1172/JCI59643, PMID: 22378047 PMC3287223

[191] Jin X Ishii H Bai Z Itokazu T Yamashita T . Temporal changes in cell marker expression and cellular infiltration in a controlled cortical impact model in adult male C57BL/6 mice. PloS One. (2012) 7. doi: 10.1371/journal.pone.0041892, PMID: 22911864 PMC3404031

[192] Loane DJ Kumar A Stoica BA Cabatbat R Faden AI . Progressive neurodegeneration after experimental brain trauma: Association with chronic microglial activation. J Neuropathol Exp Neurol. (2014) 73. doi: 10.1097/NEN.0000000000000021, PMID: 24335533 PMC4267248

[193] Donat CK Scott G Gentleman SM Sastre M . Microglial activation in traumatic brain injury. Front Aging Neurosci. (2017) 9:20. doi: 10.3389/fnagi.2017.0020 28701948 PMC5487478

[194] Wang Y Leak RK Cao G . Microglia-mediated neuroinflammation and neuroplasticity after stroke. Front Cell Neurosci. (2022) 16:980722. doi: 10.3389/fncel.2022.980722, PMID: 36052339 PMC9426757

[195] Witcher KG Bray CE Dziabis JE McKim DB Benner BN Rowe RK . Traumatic brain injury-induced neuronal damage in the somatosensory cortex causes formation of rod-shaped microglia that promote astrogliosis and persistent neuroinflammation. Glia. (2018) 66. doi: 10.1002/glia.23523, PMID: 30378170 PMC7542609

[196] Ritzel RM Doran SJ Glaser EP Meadows VE Faden AI Stoica BA . Old age increases microglial senescence, exacerbates secondary neuroinflammation, and worsens neurological outcomes after acute traumatic brain injury in mice. Neurobiol Aging. (2019) 77. doi: 10.1016/j.neurobiolaging.2019.02.010, PMID: 30904769 PMC6486858

[197] Liddelow SA Guttenplan KA Clarke LE Bennett FC Bohlen CJ Schirmer L . Neurotoxic reactive astrocytes are induced by activated microglia. Nature. (2017) 541. doi: 10.1038/nature21029, PMID: 28099414 PMC5404890

[198] MiChinaga S Koyama Y . Pathophysiological responses and roles of astrocytes in traumatic brain injury. Int J Mol Sci. (2021) 22. doi: 10.3390/ijms22126418, PMID: 34203960 PMC8232783

[199] Cieri MB Ramos AJ . Astrocytes, reactive astrogliosis, and glial scar formation in traumatic brain injury. Neural Regeneration Res. (2025) 20:973–89. doi: 10.4103/NRR.NRR-D-23-02091, PMID: 38989932 PMC11438322

[200] Burda JE Sofroniew MV . Reactive gliosis and the multicellular response to CNS damage and disease. Neuron. (2014) 81. doi: 10.1016/j.neuron.2013.12.034, PMID: 24462092 PMC3984950

[201] Zhang R Zhang J Ur Rehman A Dang L Yu X Yang W . Isolating immune cells from mouse brain and skull. J Visualized Experiments. (2024). doi: 10.3791/66861, PMID: 39141553 PMC11613012

[202] Wang J Li L Xu J Gheyret D Li K Zhang X . Neutrophil extracellular traps induce endothelial damage and exacerbate vasospasm in traumatic brain injury. Theranostics. (2025) 15:9221–39. doi: 10.7150/thno.115746, PMID: 40963908 PMC12439479

[203] Schatz DA Li L Xu J Gheyret D Li K Zhang X . CD5+ B lymphocytes in high-risk islet cell antibody-positive and newly diagnosed IDDM subjects. Diabetes. (1991) 40. doi: 10.2337/diab.40.10.1314, PMID: 1718800

[204] Alam A Thelin EP Tajsic T Khan DZ Khellaf A Patani R . Cellular infiltration in traumatic brain injury. J Neuroinflamm. (2020) 17. doi: 10.1186/s12974-020-02005-x, PMID: 33143727 PMC7640704

[205] Dwyer LJ Maheshwari S Levy E Poznansky MC Whalen MJ Sîrbulescu RF . B cell treatment promotes a neuroprotective microenvironment after traumatic brain injury through reciprocal immunomodulation with infiltrating peripheral myeloid cells. J Neuroinflamm. (2023) 20. doi: 10.1186/s12974-023-02812-y, PMID: 37259118 PMC10230748

[206] Marcet P Santos N Borlongan CV . When friend turns foe: central and peripheral neuroinflammation in central nervous system injury. Neuroimmunol Neuroinflamm. (2017) 4. doi: 10.20517/2347-8659.2017.07, PMID: 29670933 PMC5901724

[207] Li FJ Long HZ Zhou ZW Luo HY Xu SG Gao LC . System Xc–/GSH/GPX4 axis: An important antioxidant system for the ferroptosis in drug-resistant solid tumor therapy. Front Pharmacol. (2022) 13:910292. doi: 10.3389/fphar.2022.910292, PMID: 36105219 PMC9465090

[208] Braun M Vaibhav K Saad N Fatima S Brann DW Vender JR . Activation of myeloid TLR4 mediates T lymphocyte polarization after traumatic brain injury. J Immunol. (2017) 198. doi: 10.4049/jimmunol.1601948, PMID: 28341672 PMC5417078

[209] Duffy SS Keating BA Perera CJ Lees JG Tonkin RS Makker PGS . Regulatory T cells and their derived cytokine, interleukin-35, reduce pain in experimental autoimmune encephalomyelitis. J Neurosci. (2019) 39. doi: 10.1523/JNEUROSCI.1815-18.2019, PMID: 30651334 PMC6433755

[210] Biasibetti-Brendler H Schmitz F Pierozan P Zanotto BS Prezzi CA de Andrade RB . Hypoxanthine induces neuroenergetic impairment and cell death in striatum of young adult wistar rats. Mol Neurobiol. (2018) 55. doi: 10.1007/s12035-017-0634-z, PMID: 28593435

[211] Gao Y Zhang MY Wang T Fan YY Yu LS Ye GH . IL-33/ST2L signaling provides neuroprotection through inhibiting autophagy, endoplasmic reticulum stress, and apoptosis in a mouse model of traumatic brain injury. Front Cell Neurosci. (2018) 12. doi: 10.3389/fncel.2018.00095, PMID: 29922130 PMC5996884

[212] Krämer TJ Hack N Brühl TJ Menzel L Hummel R Griemert EV . Depletion of regulatory T cells increases T cell brain infiltration, reactive astrogliosis, and interferon-γgene expression in acute experimental traumatic brain injury. J Neuroinflamm. (2019) 16. doi: 10.1186/s12974-019-1550-0, PMID: 31383034 PMC6683516

[213] Thapa K Khan H Singh TG Kaur A . Traumatic brain injury: mechanistic insight on pathophysiology and potential therapeutic targets. J Mol Neurosci. (2021) 71. doi: 10.1007/s12031-021-01841-7, PMID: 33956297

[214] Kempuraj D Thangavel R Natteru PA Selvakumar GP Saeed D Zahoor H . Neuroinflammation induces neurodegeneration. J Neurol Neurosurg Spine. (2016) 1. PMC5260818 28127589

[215] Ghaith HS Nawar AA Gabra MD Abdelrahman ME Nafady MH Bahbah EI . A literature review of traumatic brain injury biomarkers. Mol Neurobiol. (2022) 5. doi: 10.1007/s12035-022-02822-6, PMID: 35499796 PMC9167167

[216] O’Brien WT Pham L Symons GF Monif M Shultz SR McDonald SJ . The NLRP3 inflammasome in traumatic brain injury: Potential as a biomarker and therapeutic target. J Neuroinflamm. (2020) 17. doi: 10.1186/s12974-020-01778-5, PMID: 32252777 PMC7137518

[217] Ravula AR Murray KE Rao KVR Pfister BJ Citron BA Chandra N . MCC950 attenuates microglial NLRP3-mediated chronic neuroinflammation and memory impairment in a rat model of repeated low-level blast exposure. J Neurotrauma. (2024) 41. doi: 10.1089/neu.2023.0444, PMID: 38269433

[218] Vincent JC Garnett CN Watson JB Higgins EK Macheda T Sanders L . IL-1R1 signaling in TBI: assessing chronic impacts and neuroinflammatory dynamics in a mouse model of mild closed-head injury. J Neuroinflamm. (2023) 20. doi: 10.1186/s12974-023-02934-3, PMID: 37884959 PMC10601112

[219] Bieniek KF Ross OA Cormier KA Walton RL Soto-Ortolaza A Johnston AE . Chronic traumatic encephalopathy pathology in a neurodegenerative disorders brain bank. Acta Neuropathol. (2015) 130. doi: 10.1007/s00401-015-1502-4, PMID: 26518018 PMC4655127

[220] Fesharaki-Zadeh A Datta D . An overview of preclinical models of traumatic brain injury (TBI): relevance to pathophysiological mechanisms. Front Cell Neurosci. (2024) 18. doi: 10.3389/fncel.2024.1371213, PMID: 38682091 PMC11045909

[221] Chung DeC Roemer S Petrucelli L Dickson DW . Cellular and pathological heterogeneity of primary tauopathies. Mol Neurodegeneration. (2021) 16. doi: 10.1186/s13024-021-00476-x, PMID: 34425874 PMC8381569

[222] Ding X Cao S Wang Q Du B Lu K Qi S . DNALI1 Promotes Neurodegeneration after Traumatic Brain Injury via Inhibition of Autophagosome-Lysosome Fusion. Advanced Sci. (2024) 11. doi: 10.1002/advs.202306399, PMID: 38348540 PMC11022701

[223] Nakamura T Lipton SA . Nitric oxide-dependent protein post-translational modifications impair mitochondrial function and metabolism to contribute to neurodegenerative diseases. Antioxidants Redox Signaling. (2020) 32:817–33. doi: 10.1089/ars.2019.7916, PMID: 31657228 PMC7074890

[224] Hu M Zhu D Zhang J Gao F Hashem J Kingsley P . Enhancing endocannabinoid signalling in astrocytes promotes recovery from traumatic brain injury. Brain. (2022) 145. doi: 10.1093/brain/awab310, PMID: 35136958 PMC8967103

[225] Li G Iliff J Shofer J Mayer CL Meabon J Cook D . CSF β-amyloid and tau biomarker changes in veterans with mild traumatic brain injury. Neurology. (2024) 102. doi: 10.1212/WNL.0000000000209197, PMID: 38478804

[226] Fordington S Manford M . A review of seizures and epilepsy following traumatic brain injury. J Neurol. (2020) 267. doi: 10.1007/s00415-020-09926-w, PMID: 32444981 PMC7501105

[227] Kumar P Lim A Hazirah SN Chua CJH Ngoh A Poh SL . Single-cell transcriptomics and surface epitope detection in human brain epileptic lesions identifies pro-inflammatory signaling. Nat Neurosci. (2022) 25. doi: 10.1038/s41593-022-01095-5, PMID: 35739273 PMC9276529

[228] Sharma R Leung WL Zamani A O’Brien TJ Espinosa PMC Semple BD . Neuroinflammation in post-traumatic epilepsy: Pathophysiology and tractable therapeutic targets. Brain Sci. (2019) 9. doi: 10.3390/brainsci911031, PMID: 31717556 PMC6895909

[229] Diamond ML Ritter AC Failla MD Boles JA Conley YP Kochanek PM . IL-1β associations with posttraumatic epilepsy development: A genetics and biomarker cohort study. Epilepsia. (2015) 56. doi: 10.1111/epi.13100, PMID: 26149793

[230] Webster KM Sun M Crack P O’Brien TJ Shultz SR Semple BD . Inflammation in epileptogenesis after traumatic brain injury. J Neuroinflamm. (2017) 14. doi: 10.1186/s12974-016-0786-1, PMID: 28086980 PMC5237206

[231] Proescholdt MG Chakravarty S Foster JA Foti SB Briley EM Herkenham M . Intracerebroventricular but not intravenous interleukin-1β induces widespread vascular-mediated leukocyte infiltration and immune signal mRNA expression followed by brain-wide glial activation. Neuroscience. (2002) 112. doi: 10.1016/S0306-4522(02)00048-9, PMID: 12074914

[232] Shaftel SS Carlson TJ Olschowka JA Kyrkanides S Matousek SB O’Banion MK . Chronic interleukin-1β expression in mouse brain leads to leukocyte infiltration and neutrophil-independent blood-brain barrier permeability without overt neurodegeneration. J Neurosci. (2007) 27. doi: 10.1523/JNEUROSCI.1418-07.2007, PMID: 17728444 PMC6673122

[233] Balosso S Liu J Bianchi ME Vezzani A . Disulfide-containing high mobility group box-1 promotes N-methyl-d-aspartate receptor function and excitotoxicity by activating toll-like receptor 4-dependent signaling in hippocampal neurons. Antioxid Redox Signal. (2014) 21. doi: 10.1089/ars.2013.5349, PMID: 24094148

[234] Maroso M Balosso S Ravizza T Liu J Aronica E Iyer AM . Toll-like receptor 4 and high-mobility group box-1 are involved in ictogenesis and can be targeted to reduce seizures. Nat Med. (2010) 16. doi: 10.1038/nm.2127, PMID: 20348922

[235] Gao W Li F Zhou Z . IL-2/Anti-IL-2 complex attenuates inflammation and BBB disruption in mice subjected to traumatic brain injury. Front Neurol. (2017) 8., PMID: 28713327 10.3389/fneur.2017.00281 PMC5492331

[236] Selvakumar GP Ahmed ME Iyer SS . Absence of glia maturation factor protects from axonal injury and motor behavioral impairments after traumatic brain injury. Exp Neurobiol. (2020) 29:230., PMID: 32565489 10.5607/en20017 PMC7344375

[237] Massagué J Sheppard D . TGF-β signaling in health and disease. Cell. (2023) 186:4007–4037., PMID: 37714133 10.1016/j.cell.2023.07.036 PMC10772989

[238] Tzavlaki K Moustakas A . TGF-β Signaling. Biomolecules. (2020) 10:487. 32210029 10.3390/biom10030487 PMC7175140

[239] Sîrbulescu RF Chung JY Edmiston WJ . Intraparenchymal application of mature B lymphocytes improves structural and functional outcome after contusion traumatic brain injury. J Neurotrauma. (2019) 36:25797–2589., PMID: 30997843 10.1089/neu.2018.6368

